# Extensive photophysiological variation in wild barley is linked to environmental origin

**DOI:** 10.1111/nph.70659

**Published:** 2025-11-11

**Authors:** Matthieu Breil‐Aubert, Katie Shaw, Jessica Royles, Cristina R. G. Sales, Julia Walter, Georgia Taylor, Richard L. Vath, Eyal Bdolach, Lalit D. Tiwari, Jyotirmaya Mathan, Tracy Lawson, Eyal Fridman, Johannes Kromdijk, John N. Ferguson

**Affiliations:** ^1^ Department of Plant Sciences University of Cambridge Cambridge CB2 3EA UK; ^2^ Plant Sciences Institute, Volcani Center Agricultural Research Organization (ARO) Bet Dagan 5025001 Israel; ^3^ School of Life Sciences University of Essex Colchester CO4 3SQ UK

**Keywords:** GWAS, local adaptation, photosynthesis, stomatal conductance, wild barley

## Abstract

- Intraspecific variation between crop wild relatives (CWRs) represents a source of untapped genetic diversity for crop improvement. At the same time, improving photosynthesis in crops has the potential to enhance yield. Thus, exploring variation for photophysiology within CWRs is an important, yet underexplored, research area.
- We describe a common garden experiment where 320 wild barley accessions were grown across two seasons. A photophysiology phenotyping pipeline was employed to quantify > 30 traits within this diversity panel. Population genetics, genome‐wide association analyses (GWAS) and deep phenotyping were performed to address local adaptation hypotheses.
- Heritable variation was detected across this photophysiological spectrum, with genotype‐by‐environment (G × E) interactions being prevalent. Evidence for local adaptation was observed in the form of subpopulation differences, signals of selection and allele frequency variation associated with markers identified via GWAS. Phenotyping of representative accessions across distinct water availabilities highlighted a role for stomatal conductance (*g*_s_) in adaptation to dry environments.
- We identified substantial variation in key photosynthesis‐associated traits in a CWR closely related to barley, an economically important crop species. Our results demonstrate that this variation is partially due to local adaptation, where plasticity in *g*_s_ appears important for maintaining photosynthesis and biomass accumulation in water‐restricted conditions.

## Introduction

Genetic gain for yield in barley ranges between 0.43% and 1.07% per annum (Cossani *et al*., 2022; Åstrand *et al*., 2024; Giménez *et al*., 2024). These rates of yield increases will be insufficient to meet future demands, especially considering the challenges posed by climate change (Åstrand *et al*., 2024). Consequently, breeding barley varieties for improved yields that are stable in the face of reduced resource inputs and environmental stress is a key priority for future food security (Jiang *et al*., 2025). Photosynthetic conversion efficiency is a major determinant of yield potential (Monteith *et al*., 1997); thus, improving photosynthesis has become increasingly recognised as a viable target for increasing crop yields (Long *et al*., 2006; Zhu *et al*., 2010). Across the major non‐leguminous C_3_ species, this conversion efficiency is lowest on average in barley (Slattery & Ort, 2015). There are several strategies available to alleviate associated conversion inefficiencies (reviewed by Croce *et al*., 2024). One approach is to explore natural variation of photosynthesis as a source of novel genes and alleles that could help fine‐tune photosynthesis to specific environmental conditions and enhance yield (reviewed by Theeuwen *et al*., 2022). To this end, it is pertinent to consider the work of Gao *et al*. (2024) who evaluated multiple photosynthesis‐related parameters via chlorophyll fluorescence across 23 spring barley varieties. Here, traits that define the rate at which atmospheric CO_2_ is assimilated demonstrated moderate‐to‐high heritability. The presence of a discernible genetic basis to these traits highlights their amenability to breeding. Moreover, Gao *et al*. (2024) observed certain photosynthesis‐associated parameters were significantly correlated with yield, providing evidence to support the notion that improving photosynthesis in barley could also improve yield.

Although the ancestors of domesticated barley evolved in harsh environments, modern barley has largely been bred and cultivated in agronomically managed environments (Jiang *et al*., 2025). This is in stark contrast to crop wild relatives (CWRs) that have not passed through a domestication bottleneck and persist across environmentally challenging habitats (Tanksley & McCouch, 1997). As a result, CWRs maintain much higher degrees of genetic variability between distinct ecotypes than their domesticated relatives and have been challenged in much harsher environments over thousands of years (Zhang *et al*., 2017). Consequently, CWRs represent an untapped resource of genetic variation for improving photosynthesis under environmentally challenging situations.

There are excellent opportunities to leverage the variation that exists in CWRs to improve photosynthesis in domesticated barley. For example, the recently published pangenome of barley incorporates 23 wild barley genomes (Jayakodi *et al*., 2024), which allows identification of structural variants that have been lost due to domestication. Additionally, there are two established and well‐sequenced diversity collections of wild barley that permit genome‐wide association studies (GWAS) to identify marker–trait associations (Prusty *et al*., 2021; Sallam *et al*., 2024).

The Barley 1 K (B1K) collection of over 1000 wild barley accessions (first described in Hübner *et al*., 2009) is an ideal model system for studying local adaptation. Distinct genetic clusters exist among these accessions, where there is minimal gene flow due, in part, to geographic barriers. Environmental variables have been shown to explain a significant proportion of the genetic variation across subsets of the B1K (Hübner *et al*., 2009; Chang *et al*., 2022). These existing studies serve as an encouraging precursor for studying local adaptation and natural variation of photosynthesis within wild barley as a CWR.

Local adaptation is a widely recognised phenomenon (reviewed by Hereford, 2009), but the traits and genes involved are often unknown. To this end, there have been very few studies that have explored the role photosynthesis plays in local adaptation. Moreover, these studies tend to be concentrated on the model species Arabidopsis (*Arabidopsis thaliana*). For example, Elfarargi *et al*. (2023) demonstrated that a population of Arabidopsis which colonised an island characterised by fog‐based precipitation had much higher rates of stomatal conductance (*g*_s_) to promote local adaptation to humid conditions via an anisohydric strategy. Taking a different approach, Oakley *et al*. (2018) observed that a quantitative trait loci (QTL) regulating the photosynthetic response to cold stress also regulated local adaptation measured as reproductive fitness.

The study presented in this paper extends the above‐described approaches by showcasing the most comprehensive screening of photosynthesis across natural accessions of a CWR. Utilising 320 accessions from the B1K diversity collection, we reveal extensive, heritable variation across traits defining light‐saturated photosynthesis, the response of photoprotection and PSII quantum yields to dynamic irradiance and limitations to light‐saturated photosynthesis. We provide evidence to suggest that this variation is partially a result of differential selection across the various subpopulations for differing photophysiological properties. Specifically, we highlight how *g*_s_ may play a key role in facilitating local adaptation to drier environments. More broadly, this research showcases that extensive variation in photosynthesis is present in wild barley and that some of this variation may be greater than what has been demonstrated in domesticated barley (Gao *et al*., 2024).

## Materials and Methods

### Plant material and common garden experiments

This study incorporated 320 accessions from the B1K collection that are genetically representative of the original 1020 accessions comprising the collection (Elfarargi *et al*., 2023). Common garden experiments incorporating these accessions were carried out in 2021 and 2022 at the National Institute of Agricultural Botany (Cambridge, UK). The sites of the two common garden experiments were *c*. 870 m apart (Supporting Information Fig. S1). In 2021, all 320 accessions were included (Table S1). In 2022, 270 accessions of the original 320 were included (Table S1). Alpha lattice twice‐replicated designs were employed for the common gardens such that each accession was represented by two plots. Each replicate of the experiment consisted of eight blocks of 40 plots in 2021 and six blocks of 45 plots in 2022. Each plot was separated by 30 cm from adjacent plots. In both years, plots consisted of four rows. The outer two rows were made of drilled spring barley (cv Laureate), with the two inner rows being hand‐transplanted wild barley. Rows were spaced 10 cm apart and were 40 cm long. Each inner row of wild barley consisted of eight transplanted plants. Before transplanting, the wild barley was hand‐sown in modular trays of M2 potting compost for germination in an ambient temperature glasshouse. Following germination, the wild barley was then moved into a vernalisation room set to 5°C for 1 month. Before transplanting, the plants were moved outside to acclimate to outside conditions. Transplanting into the common garden occurred in late April. Precise dates of drilling, sowing, vernalisation and transplanting are provided in Table S2.

### Phenotyping pipeline

We developed a high‐throughput phenotyping pipeline to screen a multitude of traits (Fig. S2). All phenotyping was performed within 10 d of heading date. Heading date was scored daily on a plot‐by‐plot basis as the emergence of the spike out of the flag leaf sheath (Zadoks *et al*., 1974). A plot was considered to be heading if > 50% of the plants within that plot were heading. Days to heading (DTH) was calculated as the days between the dates of transplanting and heading on a plot basis.

Three plants were phenotyped per plot, with plants from each plot phenotyped on the same day. Plants to be phenotyped were flagged with a barcoded tag. On the day before phenotyping, flagged plants were cut at the base of the main stem, with the cut end immediately placed into water. Excised stems were then returned to the laboratory and recut under water into individual 10‐ml centrifuge tubes to maintain the water column. Stem harvesting was completed between 15:00 and 17:00 h, after which the excised stems were left on the laboratory bench at room temperature overnight. Phenotyping proceeded the next day at 06:30 h and continued until 14:30 h. We have previously shown that phenotyping performed on excised stems in barley generates data that are comparable to phenotyping leaves attached to the plant (Ferguson *et al*., 2023).

Light‐saturated gas exchange of the penultimate leaf was measured using LI‐6400XT infra‐red gas analysers (Li‐Cor Inc., Lincoln, NE, USA) equipped with 6400‐40 leaf chamber fluorometer LED light sources. Before measuring gas exchange, leaves were light‐acclimated under a series of LED panels for 30 min. The LED panels were set to a saturating irradiance of 1800 μmol m^−2^ s^−1^ photosynthetically active radiation (PAR) at leaf level to match the PAR setpoint in the leaf chambers of the infra‐red gas analysers. The remaining conditions in the leaf chambers were set as follows: 25°C block temperature, 400 μmol s^−1^ air flow, 65–75% relative humidity (RH) and 400 μmol mol^−1^ reference CO_2_ concentration. Once moved from under the LED panels and into the leaf chambers, gas exchange was logged every 10 s for 15 min. The mean values from the last 2 min were taken for light‐saturated photosynthesis (*A*_sat_) and stomatal conductance to water vapour (*g*_s_), which were also used to calculate intrinsic water‐use efficiency (iWUE) as *A*_sat_/*g*_s_. In 2022, we additionally performed a *mini* light response (*A*–*Q*) curve following this measurement of light‐saturated gas exchange. Here, a program was immediately initiated after the 15 min of high light to incrementally drop the light intensity in the following steps: 1800, 1100, 500, 300, 150 and 50. Two minutes after each light step, photosynthesis was logged before dropping to the next light level.

A randomly selected subset of accessions (Table S1) was selected for phenotyping the response of photosynthesis to changes in the intracellular concentration of CO_2_ (*A*–*C*_i_ curve). All accessions used for *A*–*C*_i_ curves in 2021 were also used in 2022, with some additional accessions added in 2022. These measurements were performed using LI‐6800 infra‐red gas analysers equipped with a standard 6 cm^2^ leaf chamber (Li‐Cor Inc.) with environmental conditions set as follows: 25°C temperature (heat exchanger), 400 μmol s^−1^ air flow, 65% relative humidity (RH), 400 μmol mol^−1^ reference CO_2_ concentration and 1800 μmol m^−2^ s^−1^ PAR. Once stomatal conductance and photosynthesis were stable, an *A*–*C*_i_ curve program was initiated to log rates of gas exchange at the following CO_2_ reference concentrations: 400, 300, 200, 100, 50, 400, 400, 700, 1000, 1300 and 1800 μmol mol^−1^, waiting between 1.5 and 3 min between each CO_2_ step depending on standard stability criteria.

Following gas exchange phenotyping, the penultimate leaf was excised and photographed. The leaf was then carefully folded into a coin envelope and placed into a drying oven set at 60°C for 7 d. The photographs of the leaves were used to measure leaf area using Easy Leaf Area (Easlon & Bloom, 2014). Once the leaves were fully dried, we calculated specific leaf area as the ratio of the leaf area to dry mass.

Finally, *c*. 3 × 1 cm strip of tissue from the flag leaf (all other phenotyping performed on the penultimate leaf) was cut and used for chlorophyll fluorescence in a FluorCam closed chlorophyll fluorescence imaging system (Photon Systems Instruments, Brno, Czechia) exactly as described previously (Ferguson *et al*., 2025). We measured the quantum efficiency of PSII (*F*_v_/*F*_m_) as well as the response of non‐photochemical quenching (NPQ) and photosystem II (PSII) operating efficiency (ΦPSII) to an actinic light (1800 μmol m^−2^ s^−1^) being switched on for 600 s and then off for 800 s.

An *c*. 3 × 1.5 cm strip of tissue was excised from the dried leaves used to calculate SLA and ground in a bead mill. The ground, dried tissue was precisely weighed (0.5 mg ± 10%) into individual tin capsules and underwent analysis for leaf carbon (%C) and leaf nitrogen (%N) as a percentage of dried mass, as well as carbon isotope composition (δ^13^C) and nitrogen isotope composition (δ^13^N). These measurements were carried out using a Costech Elemental Analyser attached to a Thermo DELTA V mass spectrometer (Thermo Fisher Scientific Inc.) via Conflo IV in continuous flow mode.

### Photosynthesis modelling

The light‐response (*A*_N_–*Q*) data generated in 2022 were fitted using the custom fit_AQ_curve() function available on the ‘AQ_curves’ Github repository (Tomeo, 2024). This function uses the non‐rectangular hyperbola model described in Lobo *et al*. (2013). From this, we obtained estimates for respiration in the light (*R*_L_) and the apparent maximum quantum yield (ΦCO_2max_).

The CO_2_‐response (*A*–*C*_i_) data were fitted according to the FvCB model (Farquhar *et al*., 1980) using the fitacis() function from the plantecophys R package (Duursma, 2015). We used the bilinear method to estimate transition points. From this, estimates of the maximum rate of Rubisco carboxylation (*V*_cmax_), the maximum rate of electron transport for RuBP regeneration (*J*_max_) and triose phosphate utilisation (TPU) limitation were obtained on a *c*_i_ basis. We additionally estimated the stomatal limitation (SL) on photosynthesis following Long & Bernacchi (2003).

We used linear and exponential models to describe the induction of NPQ in response to the actinic light being switched on. We also used an exponential model to describe the relaxation of NPQ and recovery of ΦPSII in response to the actinic light being switched off. We have described these models previously (Ferguson *et al*., 2025). These models allowed us to determine the following: the slope of the initial induction of NPQ (NPQ_linear_); the amplitude (NPQ_ind‐amp_) and rate (NPQ_ind‐rate_) of NPQ induction; the amplitude (NPQ_rel‐amp_), rate constant (NPQ_rel‐rate_) and model offset (NPQ_rel‐res_) of NPQ relaxation; the amplitude (ΦPSII_rec‐amp_), rate (ΦPSII_rec‐rate_) and model offset (ΦPSII_rec‐res_) of ΦPSII recovery.

### Statistical analyses

Unless stated, all data handling and statistical analyses were performed within R (R Core Team, 2021) using ggplot2 (Wickham, 2009) for graphing.

We used the H2cal() function from the inti R package (Lozano‐Isla, 2024) to generate breeding values (Best Linear Unbiased Predictors (BLUPs) and Best Linear Unbiased Estimators (BLUEs)). Mixed models were constructed that incorporated genotype, block, column, replicate, and days post heading as fixed (BLUPs) or random (BLUEs) predictors of trait values on a year‐by‐year basis. We also produced joint‐year models that include interactions between these predictors and year. The variance components from these models were used to calculate broad‐sense heritability (*H* ^2^) according to Cullis *et al*. (2006) and Piepho & Möhring (2007). Piepho and Möhring estimated heritability was defined as follows: $H_{\mathrm{PM}}^{2}=1-\left(\mathrm{PE}\bar{V}/\sigma_{g}^{2}\right)$. Here, $\mathrm{PE}\bar{V}$ represents the mean prediction error variance of the genotypic BLUPs and $\sigma_{g}^{2}$ represents the estimated genotypic variance from the mixed model. Cullis estimated heritability was defined as follows: $H_{C}^{2}=1-\left(\mathrm{PE}\bar{V}/\left(2\sigma_{g}^{2}\right)\right)$. Here, $\mathrm{PE}\bar{V}$ represents the mean prediction error variance of the genotypic BLUPs and $\sigma_{g}^{2}$ represents the estimated genotypic variance from the mixed model.

Pairwise trait correlations and correlations between the same traits across years were examined via the Pearson correlation coefficient (*r*) and associated *P*‐values (*P*) using the cor() base R function. Significant correlations were defined as those where *P <* 0.05. Differences in traits and bioclimatic parameters between subpopulations were tested via one‐way analysis of variance (ANOVA) comparison of means testing using the aov() function in R. Significant differences between subpopulations were defined as those where *P <* 0.05. *Post hoc* Tukey tests were performed to determine which subpopulations were significantly different from one another. This was achieved using the HSD.test() function from the agricolae R package (Mendiburu & Simon, 2015). Bioclimatic parameters (Fick & Hijmans, 2017) were obtained for the point of collection of all accessions using the extract() function from the terra R package (Hijmans, 2025).

A quantitative estimate of phenotypic plasticity (G × E) was estimated by calculating the difference in trait values for each accession between field years, weighted by the average population value for each respective year. This was computed using the formula: (Population Mean 2021 + BLUE accession value 2021) − (Population Mean 2022 + BLUE accession value 2022). This approach captures the extent of phenotypic variation in response to environmental differences between years.

### Population genetics

A previously published customised SNP genotyping dataset for wild barley (Tiwari *et al*., 2024) was used for population genetics and genome‐wide association analyses.

For phylogenetic analyses, we first converted the VCF format of the previously described SNP dataset to a ‘genind’ object using the loci2genind function from the pegas package (Paradis, 2010). We then calculated a neighbour‐joining tree (dendrogram) with bootstrap support based on Nei's distance, using the aboot function from the poppr package (Kamvar *et al*., 2015). Finally, we extracted the genotype order from the tree to properly align and sort the structure plot.

For Structure analyses, we first converted the VCF format to a numeric matrix using a custom function. To estimate the optimal number of subpopulations (dimensions), we used the estimate_d function from the alstructure package (Cabreros & Storey, 2019), which is based on the method from (Leek, 2011). We then applied the alstructure function from the same package to compute global ancestry estimates under the admixture model, utilising the ALStructure algorithm (Cabreros & Storey, 2019). A genotype was assigned to a subpopulation if it had an admixture proportion > 50% in one ‘cluster’ (subpopulation).

We performed analyses to detect signals of natural selection using the driftsel R package (Karhunen *et al*., 2013). driftsel is a Bayesian method that compares predicted and observed mean additive genetic values to generate the *S* statistic, which indicates whether population divergence is driven by divergent selection (*S* ~ 1), stabilising selection (*S* ~ 0), or genetic drift (intermediate *S* values). This method is particularly effective for small datasets and can differentiate between drift and selection even when *Q*_ST_ (divergence in quantitative traits) and *F*_ST_ (divergence in neutral molecular markers) are equal, assuming that phenotypic variation is determined by additive genotypic variation. To estimate the coancestry coefficient matrix, we used the rafm package (Karhunen & Ovaskainen, 2012). Both the rafm and driftsel models were fit using 15 000 Markov chain Monte Carlo (MCMC) iterations, with the first 5000 iterations discarded as burn‐in and the remaining samples thinned by a factor of 2, resulting in 5000 posterior distribution samples. Due to a lack of information on the dams and sires of the phenotyped wild barley individuals, we made a slight modification to rafm and driftsel. We adopted a conservative assumption that each genotype's dam and sire are the same and that all genotypes are unrelated.

We carried out a genome‐wide association study (GWAS) using three independent, iterative statistical models to enhance accuracy. These models included the multi‐locus mixed model (MLMM; Segura *et al*., 2012), the Bayesian‐information and Linkage‐disequilibrium Iteratively Nested Keyway (blink; Huang *et al*., 2019) and the Fixed and Random Model Circulating Probability Unification (FarmCPU; X. Liu *et al*., 2016). All analyses were performed in R using gapit v.3 (Wang & Zhang, 2021), applied to both BLUEs and BLUPs. These GWAS models are described in the associated references, but all included cofactors to account for population structure (three principal components) and kinship. To prioritise ‘high‐confidence’ marker–trait associations, herein termed quantitative trait loci (QTL), we retained only those identified with both BLUEs and BLUPs across a joint‐years model for further analysis. The genome‐wide statistical significance threshold for all methods was set using the Bonferroni correction (α = 0.05), which adjusts the threshold according to the number of SNPs tested.

Alongside comparing mean trait values (as mentioned in the previous section), we also compared allele frequencies of SNPs that passed the above‐described significance threshold across subpopulations. This was achieved by converting the genotype into allele frequencies with homozygous reference alleles coded as 0 and homozygous alternate alleles coded as 1.

### Targeted experiment on representative genotypes from Steppe Jerusalem and Desert Jordan subpopulations

**Fig. 1:**
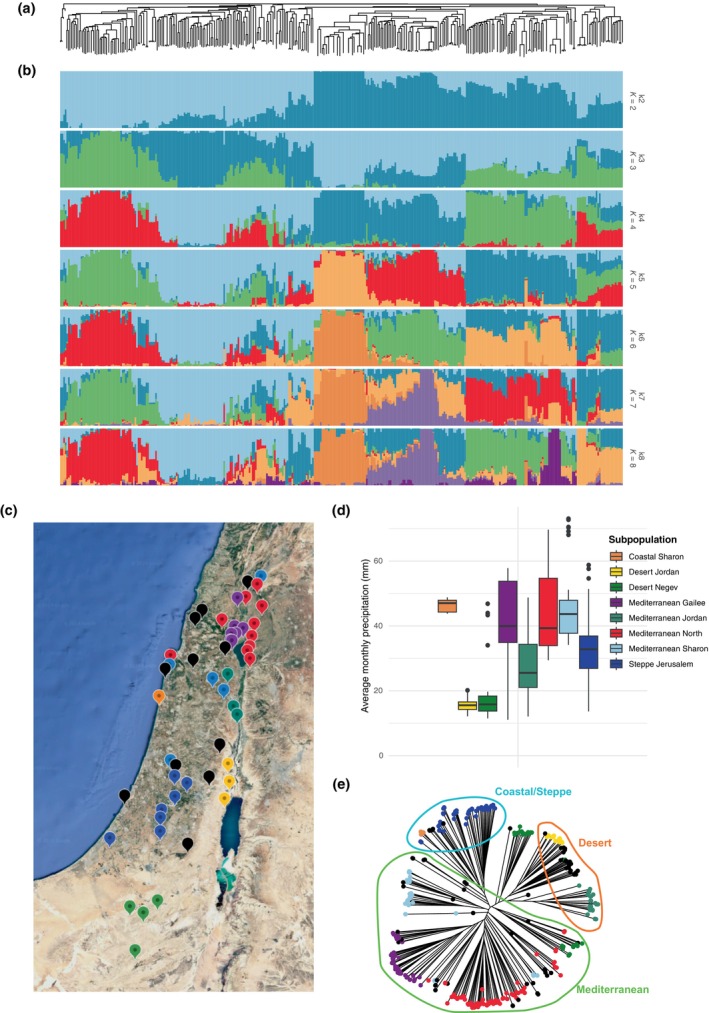
Population genetic structure of 320 wild barley (*Hordeum spontaneum*) accessions across Israel. (a) Unrooted neighbour‐joining (NJ) tree computed by Nei distance. (b) Results of the Structure analyses at *K =* 2–8. (c) Map showing the geographic sites where accessions were collected. Sites are coloured according to the Structure‐defined subpopulations that the associated accessions belong to. Multiple accessions are collected from each site, where all accessions from the same site were assigned to the same subpopulation. (d) Differences in average monthly precipitation between subpopulations. (e) Unrooted neighbour‐joining tree computed by Nei distance. Accessions are coloured according to the Structure‐defined subpopulations they belong to. Accessions are clustered into three major groups, which correspond to broad geographic areas (Mediterranean, Desert and Coastal/Steppe) as indicated by coloured ellipses.

To test hypotheses regarding the adaptive capacity to reduced water availability, we selected six accessions for phenotyping under distinct water availabilities. These accessions were selected based on principal component analyses (PCA) performed using prcomp(). The PCA biplot was visualised using fviz_eig() (Kassambara & Mundt, 2020). The traits used for the PCA were leaf area, SLA, *A*_sat_, *g*_s_, δ^13^C, δ^15^N, DTH, final NPQ, NPQ_rel‐amp_ and maximum NPQ. We selected six accessions that well‐represented the total phenotypic trait spaces (Fig. S3). Three of these accessions were assigned by our Structure analysis (Fig. 1b) to the ‘Desert Jordan’ subpopulation (B1K‐05‐12, B1K‐05‐08 and B1K‐12‐10), and three were assigned to the ‘Steppe Jerusalem’ subpopulation (B1K‐17‐17, B1K‐10‐01 and B1K‐49‐10).

Seeds from these accessions were placed in petri dishes containing damp filter paper. These were then wrapped in aluminium foil and transferred to the fridge for 6 d before moving to room temperature for 4 d. Germinated seedlings were transplanted into 1.5‐l pots containing 950 g of topsoil. Once the plants were established, a known mass of polypropylene beads was placed on top of the soil to limit evaporation from the soil. Plants were grown under glasshouse conditions (Cambridge University Botanic Garden, Cambridge, UK). A nematode biological control treatment (*Steinernema fletiae* and *Steinernema carpocapsae*; Koppert Biological Systems, Haverhill, UK) was applied to each pot on 7 d post‐transplanting to control scarid fly. Environmental conditions in the glasshouse were set as follows: 60% relative humidity (RH), 28°C day temperature, 23°C night temperature. Day length was set to 16 h. A TinyTag Ultra 2 TGU‐4500 (Gemini Data Loggers, Chichester, UK) was placed in the glasshouse for the duration of the experiment to record temperature and RH (Fig. S4). A BF5 sunshine sensor and GP1 data logger (DELTA‐T Devices, Cambridge, UK) were also placed in the glasshouse to measure incident radiation (Fig. S5).

Pots were initially well‐watered for 14 d to allow for plant establishment before being exposed to two contrasting water availability treatments. Here, the plants were either maintained at 40% relative soil water content (rSWC) or 80% rSWC, where 100% rSWC is equivalent to field capacity. Pots were maintained at target rSWCs on a daily basis as described previously (Ferguson *et al*., 2019). Eighteen soil‐only control pots were used to account for direct evaporation from the soil and these were distributed equally among the treatment pots.

*A*–*C*_i_ response measurements were performed 34–40 d post‐transplanting on recently fully expanded leaves using LI‐6400XT infra‐red gas analysers (Li‐Cor Inc.) as described previously (Ferguson *et al*., 2023). Five plants per accession per treatment were measured between 07:00 and 16:00 h. Data were processed as described in the ‘Photosynthesis modelling’ section.

At the 55^th^ day post‐transplanting, all leaves from each plant were excised and a top‐down photograph was taken to measure total leaf area using Easy Leaf Area (Easlon & Bloom, 2014).

Two‐way ANOVAs were carried out to analyse the data from our glasshouse experiment. This was performed by using the aov() function in R, with genotype nested within subpopulation, to test for statistically significant differences between rSWC treatment and subpopulation (and potential interactions). The TukeyHSD() function in R was used to determine significant pairwise interactions. shapiro.test() and leveneTest() were used to test for the normality and equal variance assumptions of ANOVA. Parameters that did not meet the normality assumptions (total leaf area and iWUE) were log‐transformed before performing further statistical analysis. Note that for total leaf area, the equal variance assumption was not met. Percentage change was determined by calculating the change in parameter mean between the 80% and 40% rSWC treatments for each subpopulation, dividing by the 80% rSWC parameter mean and multiplying by 100.

## Results

### Wild barley diversity is structured in genetic clusters across the Southern Levant that demonstrate distinct bioclimatic profiles

The population structure of the wild barley accessions incorporated as part of this study was investigated using Structure. We observed clear clustering of individual accessions into distinct subpopulations (Fig. 1a). Using a conditional factor model, we determined that the optimum number of subpopulations (*K*) was eight (Fig. 1b), with 69 accessions being classified as admixed (Table S1). In general, our analyses suggest subpopulation specificity (Evanno *et al*., 2005).

The identified subpopulations clustered geographically. Consequently, we named these subpopulations according to the regions where they were collected (Fig. 1c; Table S1): Mediterranean North (71 accessions), Mediterranean Sharon (37 accessions), Mediterranean Galilee (41 accessions), Coastal Sharon (8 accessions), Mediterranean Jordan (29 accessions), Steppe Jerusalem (48 accessions), Desert Jordan (19 accessions) and Desert Negev (33 accessions). The Mediterranean subpopulations dominate the northern and western parts of the sampling area. The Coastal and Steppe subpopulations are primarily located in transitional zones between the Mediterranean and Desert regions, whilst the Desert subpopulations are concentrated in the Southern and Eastern arid regions. Admixed individuals are predominantly found at the interfaces between these regions, suggesting genetic exchange in areas of overlapping environmental conditions.

To gauge the potential for climatic drivers of differentiation between the geographically distinct subpopulations, we obtained bioclimatic variables using the latitudinal and longitudinal coordinates associated with the point of collection of all accessions. We then compared these bioclimatic variables across subpopulations (Figs 1d, S6). Accessions aligned to the Desert subpopulations come from areas characterised by low precipitation and high temperatures. Contrastingly, the point of origin of the Mediterranean accessions tended to be much wetter and milder in temperature. The regions harbouring the Coastal and Steppe accessions occupied intermediary climatic regions. We additionally used a neighbour‐joining method (Nei's distance) to compute genetic distances and generate an unrooted phylogenetic tree of all accessions (Fig. 1e). This approach corroborated our Structure analyses and confirmed the geographic clustering of the identified subpopulations. Here, the Mediterranean subpopulations form a distinct clade that is separate from the clades that incorporate the Desert and Coastal‐Steppe subpopulations. This supports the hypothesis that genetic differentiation is driven by geographic isolation and adaptation to specific environmental conditions.

### Wild barley accessions demonstrate heritable variation for photosynthetic and life history traits

We monitored daily temperature and water inputs (precipitation and irrigation) during the 2021 and 2022 common garden experiment. The 2022 growing season was markedly warmer than the 2021 growing season (Fig. S7). Water inputs were similar between the 2 yr (Fig. S8).

**Fig. 2:**
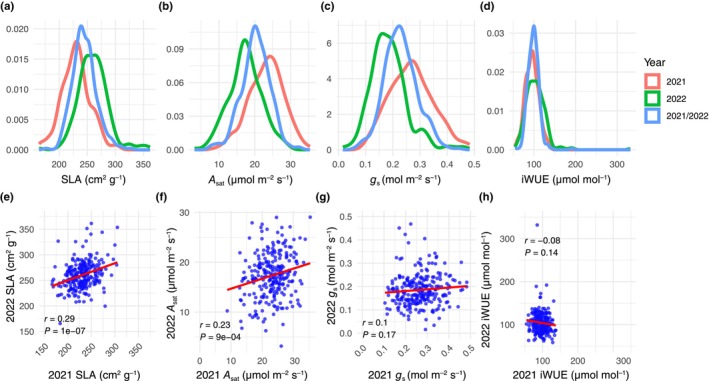
Phenotypic BLUEs variation characterised across the B1K diversity set. (a–d) Histograms showing variation for specific leaf area, photosynthesis, stomatal conductance and intrinsic water‐use efficiency. Histograms are coloured according to the models the BLUEs are derived from, that is, the 2021 model, the 2022 model, or the joint year model (2021/2022). (e–h) Correlations between specific leaf area, photosynthesis, stomatal conductance and intrinsic water‐use efficiency between 2021 and 2022 field trials.

Consistent with the warmer temperatures, we saw an overall shift towards greater specific leaf area (SLA) in 2022 than in 2021 (Fig. 2a). However, we observed a marked reduction in light‐saturated photosynthetic assimilation (*A*_sat_), which was reduced on average in 2022 than in 2021 (Fig. 2b). This was matched to the trend for stomatal conductance (*g*_s_), which was also reduced in 2022 (Fig. 2c). The overall reduction in *A*_sat_ and *g*_s_ across all accessions was similar in magnitude, which is reflected in intrinsic water‐use efficiency (iWUE) variation being similar in 2021 and 2022 (Fig. 2d).

The shift in variation for the above‐described traits across each of the common garden experiments was not consistent on an accession‐by‐accession basis, since variation for most traits was only weakly correlated across the two experiments. SLA was an exception to this general trend, where accessions that demonstrated high SLA in 2021 tended to also do so in 2022 and vice versa (Fig. 2e). The same was also true of heading date (Fig. S9), although we did note that a subset of accessions demonstrated much earlier heading in 2022 (Fig. S10), which caused the distribution to appear different when plotted (Fig. S9). In general, these results suggest that variation in growth rate (indicated by SLA; Y. Liu *et al*., 2016) and phenology was strongly influenced by underlying genetics in these two environments. This suggestion is supported by the extent of observed variation explained by the accession term in the mixed linear models used to partition variances for these traits (Table S3). Variation in *A*_sat_ was marginally correlated between the two common garden experiments (Fig. 2f). However, *g*_s_ and iWUE were not correlated at all (Fig. 2g,h), which suggests there were strong genotype‐by‐environment (G × E) interactions for water use in these accessions (Figs 2c,d, S10). Traits related to the light‐dependent photosynthetic processes demonstrated moderate correlations between the two experiments (Fig. S10).

We calculated heritability in order to estimate the extent to which the overall variation could be associated with genetic variation. Average heritability estimated according to Piepho & Möhring (2007) ($H_{\mathrm{PM}}^{2}$) using the joint‐year data was 0.37 for all traits (Table 1). The area of the penultimate leaf showed the highest $H_{\mathrm{PM}}^{2}$ (0.81). The number of DTH was less heritable (0.28). Traits associated with the light‐dependent photosynthetic reactions measured via Chl fluorescence tended to be moderately heritable (0.29–0.59), whereas leaf gas exchange‐associated traits were generally less heritable (0.01–0.49). Apart from δ^13^C (0.41), traits associated with leaf elemental composition demonstrated low heritability (0.03–0.13). Depending on the trait, $H_{\mathrm{PM}}^{2}$ estimated by the individual year models was different (Table S4). Gas exchange‐associated traits demonstrated higher heritability in general in 2021 than in 2022. Conversely, traits estimated via Chl fluorescence appeared more heritable in 2022. These findings highlight that the extent of variation imparted by the environment was different between the two growing seasons in line with substantial G × E.

**Table 1:** Piepho & Möhring broad‐sense heritability ($H_{\mathrm{PM}}^{2}$) estimated from the joint year model for all traits measured across 2021 and 2022, alongside the signal of selection (*S*) estimated for the same traits using driftsel.

Trait	$H_{\mathrm{PM}}^{2}$	*S*
%N	0.08	0.38
%C	0.13	0.39
C : N	0.05	0.41
δ^13^C	0.41	0.43
NPQ_ind‐amp_	0.34	0.46
NPQ_ind‐rate_	0.39	0.36
NPQ_rel‐amp_	0.38	0.58
NPQ_rel‐rate_	0.55	0.4
NPQ_rel‐res_	0.52	0.39
ΦPSII_rec‐amp_	0.38	0.6
ΦPSII_rec‐rate_	0.56	0.37
ΦPSII_rec‐res_	0.46	0.47
NPQ_linear_	0.59	0.34
Maximum NPQ	0.44	0.53
Final NPQ	0.54	0.38
Final ΦPSII	0.29	0.37
DTH	0.28	0.44
Leaf mass	0.72	0.43
Leaf areas	0.81	0.65
SLA	0.44	0.99
*V*_cmax_	0.49	0.84
*J*_max_	0.01	1
TPU	0.31	0.36
SL	0.14	0.34
*A*_sat_	0.34	0.64
*g*_s_	0.29	0.4
iWUE	0.01	0.59

When *S* ~ 1, this indicates divergent selection between the populations; when *S* ~ 0.5, this indicates a neutral pattern (genetic drift); and when *S* ~ 0, this indicates stabilising selection between populations.

We next estimated the signal of selection (*S*) for all traits using driftsel (Karhunen & Ovaskainen, 2012). This allowed us to test for the presence of differing evolutionary pressures across subpopulations for these traits. The average *S* value for all traits was 0.50, which is indicative of a neutral pattern of selection between subpopulations due to genetic drift (Table 1). However, a few traits did demonstrate strong evidence of divergent selection between subpopulations, where *S* exceeded 0.95. For example, SLA had an *S* value of 0.99, which may suggest that the growth rate is under strong divergent selection across subpopulations. Contrastingly, the *S* value for DTH was moderate (0.44), suggesting that any potential differences in selection for growth rates between the subpopulations may be independent of floral transitioning. Interestingly, the parameters derived from the Rubisco‐limited (*V*_cmax_) and electron transport‐limited (*J*_max_) portions of the *A*–*C*_i_ curve both demonstrated high *S*‐values.

**Fig. 3:**
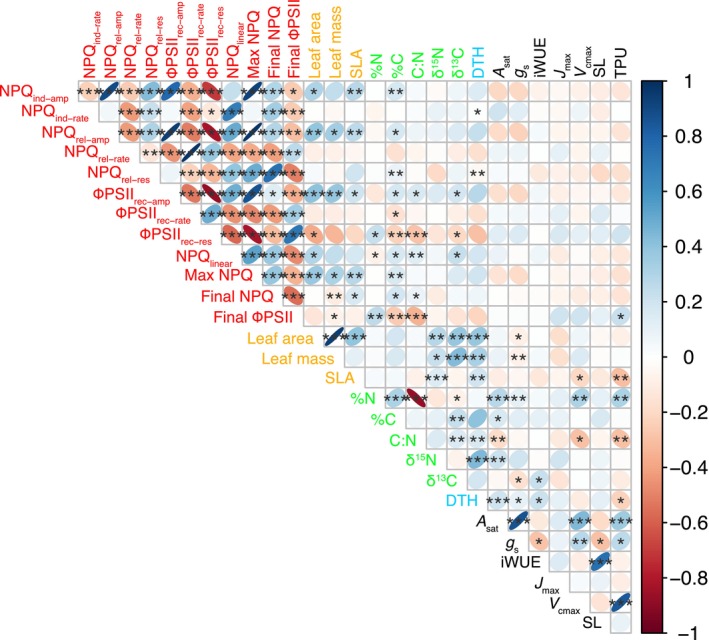
Correlogram showing pairwise trait correlations between traits (BLUEs from the joint model). Significant correlations are highlighted with asterisks at α = 0.05 (*), 0.01 (**) and 0.001 (***). The colour of the ellipses indicates the direction of the correlations. Trait names are coloured according to associated trait groupings. Red, light‐dependent reaction‐associated traits; orange, leaf structure‐associated traits; green, leaf chemical composition‐associated traits; black, gas exchange‐associated traits.

We examined correlations across all pairwise trait interactions (Figs 3, S11, S12). In general, pairwise trait correlations held true across the two common garden experiments (Figs S10, S11), with some exceptions. For example, in 2021, DTH demonstrated a strong positive correlation with *A*_sat_ and *g*_s_ (Fig. S11), suggesting that accessions that were more photosynthetically active tended to be those that had transitioned to flowering later. In 2022, conversely, *A*_sat_ was not associated with DTH (Fig. S12). Additionally, in 2022, we observed multiple significant correlations between δ^13^C and traits that relate to the response of NPQ and ΦPSII to dynamic irradiance that were not present in 2021 (Figs S10, S11). δ^13^C is a useful proxy for WUE integrated over the time in which the carbon forming the tissue was fixed (Leakey *et al*., 2019); consequently, these associations may suggest that variation in the kinetics of the light‐dependent photosynthetic reactions may have had more of a bearing on variation in WUE in 2022 than in 2021. Consistent with this assertion, we only observed a significant (negative) association between δ^13^C and *g*_s_ in 2021 but not in 2022 (Fig. S11), thereby suggesting that variation in stomatal physiology and behaviour may have been more important in defining variation in WUE in 2021. This suggestion is further supported by the presence of a significant, negative association between *g*_s_ and iWUE that was observed in 2021 only (Fig. S11).

By examining pairwise trait correlations using the joint‐year BLUEs, we were able to determine phenotypic associations that persisted despite apparent G × E interactions (Figs 1e,h, S10). Interactions of interest here included the positive correlations between the amplitude of NPQ induction and relaxation with SLA and the amplitude of the recovery of ΦPSII with SLA (Fig. 3). These associations may suggest that larger responses of these light‐dependent reaction‐associated parameters to changes in irradiance may be associated with growth rate and/or other determinants of variation in SLA. In terms of photosynthetic capacity, we observed expected associations that highlight the importance of leaf nitrogen content and *g*_s_ as determinants of *A*_sat_ (Fig. 3). Further, we observed a positive association between *V*_cmax_ and *A*_sat_, but not *J*_max_ and *A*_sat_, which suggests that variation in carboxylation by Rubisco is more important in limiting net CO_2_ assimilation than capacity for RuBP regeneration across this diversity set. Alternatively, this observation could also be a result of lighter sampling of photosynthesis at higher CO_2_ concentrations, which may limit the number of data points available to fit *J*_max_ and TPU, meaning they may be less well constrained than *V*_cmax_.

We also observed a positive association between *A*_sat_ and TPU as derived from the *A*–*C*_i_ curve (Fig. 3), which suggests that more effective export of triose phosphate is linked to higher rates of *A*_sat_ (Lombardozzi *et al*., 2018).

### Observed phenotypic variation is regulated by multiple QTL and shows evidence for local adaptation between subpopulations

For our GWAS, we adopted multiple approaches for detecting SNP–trait associations. Through these various approaches, we identified 193 QTL using the joint‐year data and 159 QTL using the individual year data. We focused on those that were identified using the joint‐year data, as these were likely to be more genetically robust. Further, we prioritised high‐confidence QTL as those that were identified using both the joint‐year BLUEs and the joint‐year BLUPs. In total, we identified 22 high‐confidence QTL that were located across all chromosomes except chromosome 6H (Table 2). Seven of these were associated with DTH, one was identified for SLA, and one was associated with the mass of the penultimate leaf. The remaining 13 QTL were linked to traits associated with light‐dependent photosynthetic processes phenotyped via Chl fluorescence. Some of these 13 QTL were non‐unique since they were identified for closely related or co‐dependent traits.

**Table 2:** High‐confidence quantitative trait loci (QTL) identified.

Chromosome	Position (bp)	–Log_10_ (*P*‐value)	Trait	GWAS detection method(s)
1H	119 640 256	5.84	DTH	FarmCPU, BLINK
1H	434 934 408	6.40	NPQ_ind‐rate_	FarmCPU, BLINK
2H	18 515 412	7.39	Maximum NPQ	FarmCPU, BLINK
2H	18 515 412	6.39	NPQ_ind‐amp_	BLINK
2H	465 880 175	10.63	DTH	FarmCPU
2H	641 328 117	5.95	DTH	FarmCPU
2H	651 770 486	5.45	NPQ_rel‐amp_	FarmCPU, BLINK
3H	358 746 713	6.68	DTH	FarmCPU
3H	578 465 223	5.64	NPQ_ind‐amp_	BLINK
3H	606 149 268	9.93	ΦPSII_rec‐rate_	FarmCPU, BLINK
4H	548 294 745	9.74	Final ΦPSII	FarmCPU, BLINK
4H	615 129 219	7.59	Final ΦPSII	FarmCPU, BLINK, mlmm
5H	565 157 996	8.73	Maximum NPQ	FarmCPU, BLINK
5H	571 285 042	5.65	SLA	BLINK
5H	588 538 716	9.09	DTH	FarmCPU, BLINK
5H	595 204 884	8.73	DTH	FarmCPU
5H	601 142 082	6.92	Maximum NPQ	BLINK
5H	603 003 010	9.32	Final ΦPSII	FarmCPU, BLINK
5H	647 383 990	11.73	NPQ_linear_	FarmCPU, BLINK
5H	647 383 990	7.62	NPQ_ind‐rate_	FarmCPU, BLINK
7H	65 422 726	8.41	Leaf mass	FarmCPU
7H	641 762 783	9.43	DTH	FarmCPU

Each row represents a significant SNP–trait association. The chromosome, basepair position, −log_10_ (*P*‐value), associated trait and method of detection are provided.

Whilst the goal of this study was not to identify candidate gene, we did explore all the genes within 100 kb (upstream and downstream) of these high‐confidence QTL. In only one instance did we observe a highly obvious associated candidate gene. This was for the QTL associated with NPQ_ind‐amp_ and maximum NPQ on chromosome 2H. Here, we observed just one gene within the 200 kb upstream and downstream window, which was *c*. 49 kb away from the SNP defining this locus and annotated as Rubisco small subunit (*rbcS*; HORVU.MOREX.r3.2HG0104730). *rbcS* mutants are known to have perturbed NPQ light responses and reduced maximum NPQ (Atkinson *et al*., 2017), thereby highlighting the plausibility of *rbcS* as a candidate gene here, although further validation would be needed.

**Fig. 4:**
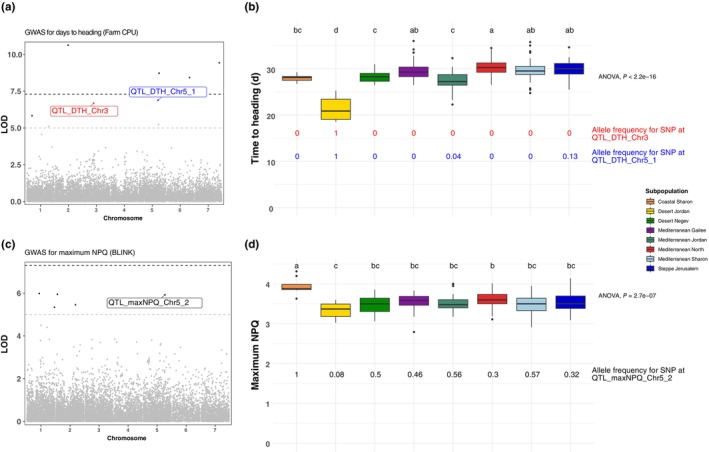
GWAS and follow‐up analyses of the allelic frequency of significant SNPs. (a) GWAS for days to heading (DTH). SNPs where allelic frequency across the subpopulations is different and mirror associated trait variation are highlighted, with those differences being demonstrated in (b). (c) GWAS for maximum non‐photochemical quenching (NPQ). A SNP whose allelic frequency across the subpopulations is different and mirrors associated trait variation is highlighted, with those differences being demonstrated in (d). Lowercase letters above boxplots in (b) and (d) represent Tukey *post‐hoc* groupings.

For all the high‐confidence QTLs we tested for differences in the allelic frequency of the SNPs associated with those QTLs between the identified subpopulations, following the approach of Fustier *et al*. (2019). Here, we identified three QTLs/SNPs where both allelic frequencies and trait variation for which the QTLs had been identified were significantly different between the subpopulations (Fig. 4). It is worth noting that only three of the 22 high‐confidence SNP–trait associations showed such a pattern, which reflects the conservative nature of our GWAS approach. Further, it reflects the fact that population structure was included as a covariate in our GWAS models, since this approach should limit the ability to detect associations where allele frequency differences coincide with population structure; however, complete correction here is inherently limited.

The QTLs identified for DTH on chromosomes 3H and 5H (1^st^ QTL) demonstrated distinct allelic frequencies with respect to the Desert Jordan subpopulation, which also demonstrated markedly earlier flowering than the remaining subpopulations (Fig. 4a,b). Given the significantly reduced precipitation that characterises the region of origin for this subpopulation (Fig. 1d), it could be suggested that there has been active selection on the alternative allele at these two QTL in the Desert Jordan subpopulation to promote early flowering as a drought adaptive mechanism.

The second QTL identified for maximum NPQ on chromosome 5H had significantly higher allelic frequency in the Coastal Sharon subpopulation, which also demonstrated significantly higher maximum NPQ than the remaining subpopulations (Fig. 4c,d). Coastal areas tend to receive more variable cloud cover, and enhanced cloudy environments have been shown to elicit higher maximum NPQ in Arabidopsis ecotypes due to a lack of adaptation to high‐light environments (Rungrat *et al*., 2019). Thus, it is plausible to suggest that the alternative allele at this QTL may be linked to an NPQ‐derived adaptation to the high light; therefore, it is selected for in the non‐Coastal Sharon accessions.

**Fig. 5:**
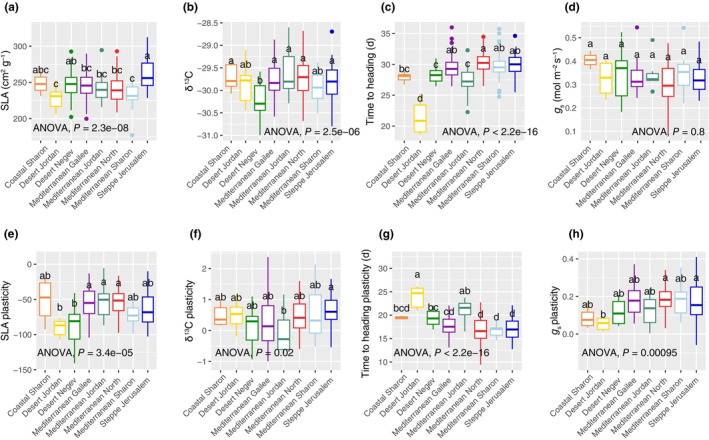
Trait variation across the subpopulations for (a) specific lear area (SLA), (b) carbon isotope composition (δ^13^C), (c) days to heading (DTH) and (d) stomatal conductance (*g*_s_). Variation in plasticity between 2021 and 2022 across the subpopulations for (e) SLA, (f) δ^13^C, (g) DTH and (h) *g*_s_. *P*‐values associated with one‐way ANOVA comparisons of means tests are inset, and associated *post hoc* classifications of the subpopulations are indicated by the letters above the boxplots. For each boxplot the horizontal line within each box represents the median; the box boundaries indicate the interquartile range (IQR) (25^th^ and 75^th^ percentile); whiskers extend 1.5 × IQR; and points beyond the whiskers represent outliers.

In order to further understand physiological mechanisms that may underpin differential adaptation across these subpopulations, we compared both absolute trait variation as well as variation in trait plasticity across the subpopulations (between the 2021 and 2022 growing seasons). Through this approach, we observed that the desert subpopulations were most commonly the outliers relative to the other subpopulations for both plasticity and absolute metrics (Figs 5, S13, S14). For example, the Desert Jordan subpopulation demonstrated significantly reduced SLA compared with all other subpopulations except the Mediterranean Sharon subpopulation (Fig. 5a). Curiously, the only subpopulation to demonstrate significantly different δ^13^C compared with other subpopulations was the Desert Negev subpopulation (Fig. 5b), which tended to show the most negative values, which would suggest reduced water‐use efficiency. This may also be a result of differential post‐photosynthetic fractionation in the Desert Negev subpopulation. The Desert Jordan subpopulation displayed by far the fastest flowering time, since DTH was significantly reduced in this subpopulation than in all other subpopulations (Fig. 5c). We did not detect any significant differences between the subpopulations for *g*_s_; however, it is notable that the Coastal Sharon subpopulation, which is characterised by the highest average monthly precipitation (Fig. 1d), had by far the highest average *g*_s_ (Fig. 5d).

With respect to differences in plasticity, the two Desert subpopulations showed the greatest change in SLA between the growing seasons, where the variation in plasticity across these two subpopulations was significantly more negative (indicating a shift towards higher SLA in 2022) than three of the four Mediterranean subpopulations (Fig. 5e). Variation in plasticity for δ^13^C was relatively consistent across the subpopulations, with only the Mediterranean Jordan and Steppe Jerusalem subpopulations showing a significant difference (Fig. 5f). Conversely, plasticity in DTH was much more variable with four *post hoc* groups identified. Here, the Desert Jordan subpopulation demonstrated the greatest plasticity (indicating a shift towards earlier flowering in 2022) with the Steppe Jerusalem subpopulation showing the least plasticity between the growing seasons (Fig. 5g). These two subpopulations were also the only two to demonstrate significantly different variation in *g*_s_ plasticity, with the Desert Jordan subpopulation showing relatively low plasticity and the Steppe Jerusalem showing comparatively higher *g*_s_ plasticity (Fig. 5h).

**Fig. 6:**
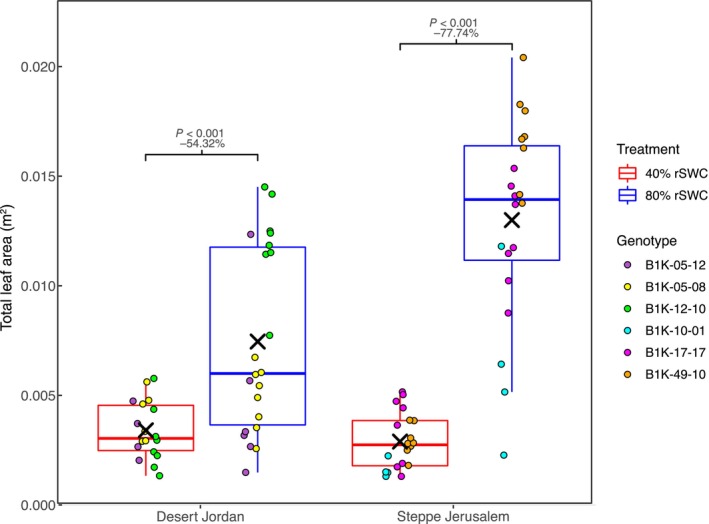
Total leaf area of the six accessions included in the detailed experiment that were subjected to two distinct watering regimes to maintain relative soil water content (rSWC) at 40% and 80%. The percentage change of the mean for each accession between 80% and 40% rSWC is inset within each subpanel alongside the associated *P*‐value obtained from *post hoc* Tukey tests applied to the associated two‐way ANOVA. For each boxplot the horizontal line within each box represents the median; the box boundaries indicate the interquartile range (IQR) (25^th^ and 75^th^ percentile); whiskers extend to 1.5 × IQR; and points beyond the IQR represent outliers.

To further test the capacity for G × E and its role in adaptation to environments across distinct subpopulations, we selected three accessions each from the Desert Jordan and Steppe Jerusalem subpopulations based on PCA (Fig. S3). These six accessions were grown under two distinct and consistently maintained water availabilities. Plants grown under reduced water availability (40% rSWC) demonstrated reduced total leaf area (Fig. 6; Table S5). This was consistent across the two subpopulations, although some genotypes demonstrated stronger reductions than others. Whilst the observation was not particularly surprising, we do note that the percentage decline was much more extreme across the Steppe Jerusalem genotypes (77.74%) relative to the Desert Jordan genotypes (54.32%).

**Fig. 7:**
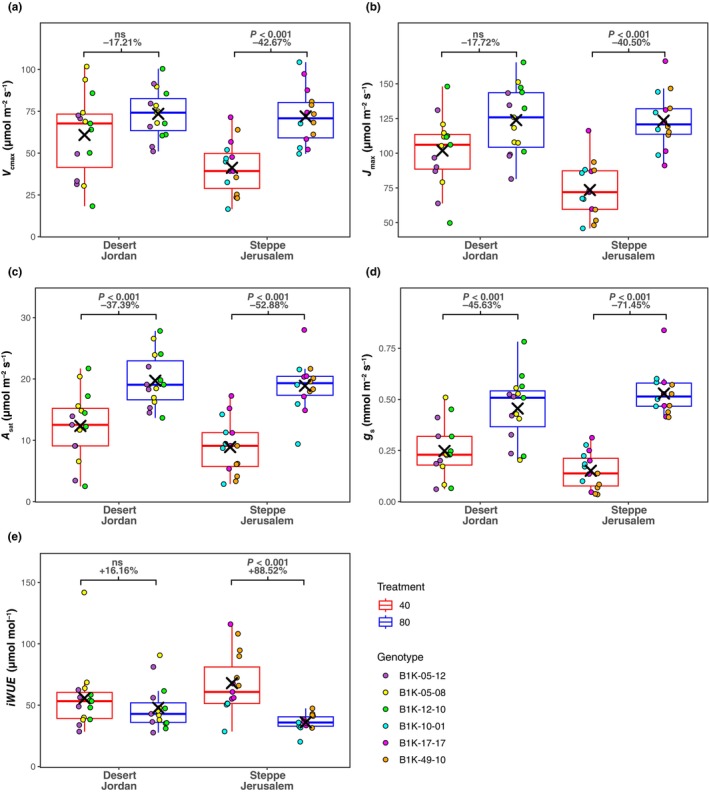
Photosynthesis‐associated traits of the six accessions included in the detailed experiment that were subjected to two distinct watering regimes to maintain relative soil water content (rSWC) at 40% and 80%. (a) Maximum rate of carboxylation by Rubisco (*V*_cmax_). (b) Maximum rate of electron transport for RuBP regeneration (*J*_max_). (c) Light‐saturated photosynthesis (*A*_sat_). (d) Stomatal conductance (*g*_s_). (e) Intrinsic water‐use efficiency (iWUE). The percentage change of the mean for each accession between 80% and 40% rSWC is inset within each subpanel alongside the associated *P*‐value obtained from *post hoc* Tukey tests applied to the associated two‐way ANOVA. For each boxplot the horizontal line within each box represents the median; the box boundaries indicate the interquartile range (IQR) (25^th^ and 75^th^ percentile); whiskers extend to 1.5 × IQR; and points beyond the IQR represent outliers.

During the above‐described water availability experiment, we profiled leaf‐level gas exchange and performed *A*–*C*_i_ response curves in order to generate data that may explain differences in biomass accumulation. Here, we observed that *V*_cmax_ and *J*_max_ were significantly reduced across the Steppe Jerusalem accessions when grown at 40% rSWC compared with those at 80% rSWC (Fig. 7a,b; Table S5). This effect was not mirrored by the Desert Jordan accessions, where neither *V*_cmax_ nor *J*_max_ was significantly reduced when associated accessions were grown at 40% rSWC (Fig. 7a,b; Table S5). *A*_sat_ was significantly reduced across the accessions from both subpopulations (Fig. 7c; Table S5), but the magnitude of the reduction was much greater across the Steppe Jerusalem accessions (52.65%) than in the Desert Jordan accessions (37.39%).

The differences in G × E for photosynthetic capacity across the two subpopulations may in part be explained by effects on *g*_s_ (Fig. 7d; Table S5). We observed that *g*_s_ was significantly reduced for both subpopulations when grown at 40% rSWC; however, this reduction was much greater (71.45%) for the Steppe Jerusalem subpopulation than for the Desert Jordan subpopulation (45.63%). This suggests that the Desert Jordan accessions are able to maintain normal photosynthetic capacity under reduced water availability despite reduced *g*_s_.

Notwithstanding the significant decline in *g*_s_, iWUE of the Desert Jordan accessions was not significantly different between the two water availability treatments (Fig. 7e; Table S5), which is further reflective of the adaptive capacity of the Desert Jordan accessions and suggests that, despite restricting water loss, there is no modulation between the amount of carbon gained per unit of water lost under the tested drought scenario. Conversely, iWUE of the Steppe Jerusalem accessions was significantly increased when plants were grown under reduced water availability (Fig. 7e; Table S5), suggesting that accessions from this subpopulation may need to fine‐tune this trade‐off relative to how they perform under optimal resource availability.

## Discussion

We presently have a limited understanding of the extent to which photosynthesis varies intraspecifically in any CWR of interest. This study addresses this for the first time through a comprehensive assessment of the natural variation of photophysiology across wild barley. We showcase extensive photosynthetic diversity for future barley improvement efforts. Below, we provide context on how this natural variation could be harnessed for crop improvement in the context of climate change.

We identified eight distinct subpopulations (Fig. 1b,c) that largely aligned with the recent work of Chang *et al*. (2022), consistent with the presence of 164 common accessions between our studies (Table S1). The relatively short geographic distances between the distinct subpopulations reflect the unique nature of the B1K collection region within Israel. Here, between relatively short distances, one can find very different environments and topographies. The apparent lack of gene flow between these subpopulations reflects previous work on the B1K that used a different marker system. This work also supported admixture between accessions in border regions (Hübner *et al*., 2012). We obtained climatic data from the point of collection of all accessions and identified distinct differences in historical water availability and growing temperatures (Figs 1d, S6). These environmental parameters are well‐known to have multifaceted effects on photosynthesis (Chaves *et al*., 2009; Yamori *et al*., 2014) and are critical in determining local adaptation (Stebbins, 1952).

We measured SLA of the penultimate leaf as an important trait for defining investment of resources and plant growth (Lambers & Poorter, 1992; Shirdelmoghanloo *et al*., 2022). SLA has been utilised in barley breeding contexts as a proxy for grain yield (Alqudah & Schnurbusch, 2015) and vigour (Rebetzke *et al*., 2004). Consequently, we utilise SLA as a proxy for biomass accumulation in our study. In alignment with previous observations in domesticated barley, we observed a strong genetic component to SLA in wild barley (Table 1). Moreover, alongside DTH (Fig. S10), SLA and its constituents showed the greatest similarity in variation across the growing seasons (Figs 2, S10), suggesting reduced G × E compared with photophysiological traits. SLA also demonstrated a high *S* value (Table 1), suggesting there is differential selection for SLA across the defined subpopulations (Fig. 1). In general, SLA increases with resource availability in the short term; however, long‐term adaptation to unfavourable habitats can also result in SLA increasing over evolutionary time despite a lack of resources (Liu *et al*., 2023). In this context, we note that accessions from the two desert environments demonstrate a significant difference in subpopulation‐wide SLA variation (Fig. 5a). Here, the Desert Jordan subpopulation demonstrated the lowest SLA, which is in line with an expected adaptation to limited water availability (Scheepens *et al*., 2010; Wilcox *et al*., 2021). However, variation in SLA across the Desert Negev subpopulation was similar to the majority of other subpopulations, suggesting that these accessions may have undergone longer term adaptation to reduced precipitation and/or there is a strong influence on SLA coming from other environmental parameters, for example, light availability (Y. Liu *et al*., 2016).

In barley, like many other species, there is a strong association between light availability and SLA (Gunn *et al*., 1999). We observed significant positive associations between SLA and traits that define the response of NPQ and ΦPSII to light (Fig. 3). Specifically, our results highlight that greater changes in the induction and relaxation of NPQ and recovery of ΦPSII are linked to greater SLA and, potentially, greater biomass accumulation. A similar observation was also made recently by Cowling *et al*. (2022), who showed that NPQ dynamics were linked to biomass accumulation across African rice landraces. We also observed differences between subpopulations for NPQ and ΦPSII dynamics (Fig. S13), highlighting how dynamic photoprotection and photosynthesis may be linked to local adaptation. Moreover, through our GWAS, we detected many marker–trait associations for these NPQ‐ and ΦPSII‐related traits (Table 2). In one case, a very obvious candidate gene was in close proximity to an identified marker. Here, *rbcS* was linked to genetic variation in the amplitude of change in NPQ upon transitioning from high light to darkness, which could relate to synchronisation between the demand for ATP for CO_2_ fixation and acidification of the thylakoid lumen (Ramakers *et al*., 2025). In another instance, we observed a link between the frequency of alleles of a marker significantly associated with maximum NPQ and subpopulation variation in maximum NPQ (Fig. 4c,d). Here, the alternative allele appeared fixed in the Coastal Sharon subpopulation, which also demonstrated significantly higher maximum NPQ than any other subpopulation. In general, coastal environments tend to be characterised by more cloud cover, which likely places significant pressure to adapt photoprotective responses to dynamic light conditions. It may be the case that Coastal Sharon accessions are not exposed to strong selection pressure to adapt to a high‐light environment, thus there is no variation in the identified allele. This would be in line with observations in Arabidopsis (Rungrat *et al*., 2019).

Similar observations were made for two markers significantly associated with variation in DTH (Fig. 4a,b). Here, the alternative allele at these two markers was dominant in the Desert Jordan subpopulation, which also had the shortest DTH of any subpopulation. This is plausibly a result of the evolution of a drought escape strategy (reviewed by Kooyers, 2015) in the Desert Jordan subpopulation. Previous common garden experiments have shown that populations from xeric environments tend to flower earlier (Knight *et al*., 2006; Lowry *et al*., 2015). As with the SLA observation described above, this trend does not hold true with the Desert Negev subpopulation, suggesting that this environment is not as stressful in terms of water availability and/or these accessions have evolved alternative drought avoidance mechanisms.

With respect to *A*_sat_, we observed a substantial amount of variation across the diversity panel (Fig. 2b) that was reasonably consistent across the two growing seasons (Fig. 2f) despite a strong contribution from the environment in determining the variation (Table 1). The heritability estimates of photosynthesis traits estimated from gas exchange in this study are reduced relative to domesticated barley (Gao *et al*., 2024). However, it is worth noting that Gao *et al*. (2024) performed measurements using plant material grown in controlled environments, thereby limiting environmental noise. In addition, domesticated barley has been specifically bred for stability (Kraakman *et al*., 2004), which is not the case for wild species (Lachowiec *et al*., 2016). Gao *et al*. (2024) observed a mean *A*_sat_ across their studied barley varieties of 17.2 μmol CO_2_ m^−2^ s^−1^ and a maximum of 19.7 μmol CO_2_ m^−2^ s^−1^. In our study, the average value for *A*_sat_ from the joint‐year BLUEs was 20.19 μmol CO_2_ m^−2^ s^−1^ and some accessions demonstrated rates exceeding 30 μmol CO_2_ m^−2^ s^−1^ (Fig. 1b). The *A*_sat_ data from our 2022 growing season were more comparable with those of Gao *et al*. (2024); however, there were still some accessions with much higher rates of *A*_sat_. Moreover, the average *A*_sat_ in 2021 was 23.31 μmol CO_2_ m^−2^ s^−1^ (Fig. 1b). In general, these results showcase that there is a wealth of variation in *A*_sat_ in wild barley. Further, these results are indicative of photosynthetic rates greater than those demonstrated in domesticated barley. Despite this finding, it is pertinent to remember that the associated phenotyping in both studies was performed under contrasting environments, which may influence the observed variation. Our work has made a start to better understand the genetics underpinning the variation in wild barley, but this will require further experimentation of derived crosses in controlled environments to maximise heritability.

Whilst we were unable to identify QTL associated with *A*_sat_ variation, we were able to assess patterns of variation that help to better understand the basis for this variation. For example, a very strong, positive association was observed with *g*_s_ (Fig. 3). This follows the well‐characterised trend that enhancing photosynthesis in a C_3_ species typically requires concurrent increases in *g*_s_ (Leakey *et al*., 2019). This clearly has implications for WUE; however, we note that the negative association between *g*_s_ and iWUE is not as strong as the *g*_s_‐*A*_sat_ association. This implies that there are some accessions that can operate at high rates of *A*_sat_ with low or moderate *g*_s_, which would be ideal for breeding barley to drought‐prone environments (Rebetzke *et al*., 2004). Indeed, our experiment with six selected contrasting accessions (discussed further below) highlights the trait combinations that could be selected for. Another commonly observed association we detected was the positive association between leaf nitrogen content and *A*_sat_ (Fig. 3), which reflects the investment in nitrogen of the Calvin Cycle and thylakoid proteins (Evans & Clarke, 2019). We also observed a significant positive association between δ^15^N and *A*_sat_ (Fig. 3). There is some evidence that carbon metabolism and nitrogen assimilation, alongside the isotopic composition of the external nitrogen source, are important in determining δ^15^N (Evans, 2001; Kalcsits *et al*., 2014). Some of the early work to understand genotypic differences in δ^15^N comes from wild barley (Handley *et al*., 1997; Robinson *et al*., 2000), where genotypic variation in δ^15^N was linked to abiotic stress tolerance and also the capacity to retain N, which would have implications for photosynthesis. This may also reflect the association between leaf δ^15^N and DTH (Fig. 3), since floral transitioning is typically associated with the remobilisation of nitrogen and the onset of senescence (Distelfeld *et al*., 2014). The importance of leaf nitrogen is further reflected in the positive correlations between *V*_cmax_ and %N and *A*_sat_, which reflects the investment of N in Rubisco (Luo *et al*., 2021) and confirms that Rubisco activity is key for photosynthetic performance in barley.

As a final component to this study, we sought to understand how differences in photophysiological plasticity may contribute to adaptation in wild barley. To this end, we focused on accessions from the Desert Jordan and the Steppe Jerusalem subpopulations as the only two groups to show differences in *g*_s_ plasticity (Fig. 5d). Plants grown under reduced water availability showed reduced total leaf area across both subpopulations (Fig. 6). This is a fairly well‐characterised response to water deficits in plants and has also been observed recently in domesticated barley (Moualeu‐Ngangué *et al*., 2020). In general, this response reflects (1) an attempt by the plant to reduce surface area for transpiration; and/or (2) a reallocation of resources to promote root growth for water acquisition (Chaves *et al*., 2009). Interestingly, the clearest difference between populations was observed under the 80% rSWC treatment, where Steppe Jerusalem accessions developed markedly larger leaf area than Desert Jordan accessions. This pattern suggests that genetic divergence in growth strategy is most evident under moderate‐to‐high water availability, whereas severe drought imposes a shared physiological limitation. Such genotype‐by‐environment interactions imply that adaptive differentiation may be more strongly linked to performance under favourable conditions, potentially reflecting selection for resource‐use efficiency in mesic environments. This hypothesis is in line with a recent study that focused on a wild C_4_ grass, *Bouteloua gracilis*, where populations from more arid environments tended to be smaller whilst demonstrating reduced plasticity to water limitation (Bushey *et al*., 2023).

The relative maintenance in vegetative biomass accumulation in Desert Jordan accessions compared with Steppe Jerusalem accessions might be related to their ability to maintain photosynthetic capacity under reduced water availability (Fig. 7a–c). Indeed, recent work on wild relatives of wheat has shown that maintaining photosynthesis under drought stress is key to maintaining biomass accumulation (Mahmood *et al*., 2023). Given the link between *g*_s_ and photosynthetic capacity (Paillassa *et al*., 2020), we were surprised to see that *g*_s_ significantly declined in the Desert Jordan accessions as well as the Steppe Jerusalem accessions (Fig. 7d). Again, however, the magnitude of this response was far greater for the Steppe Jerusalem accessions. In light of this observation and given the previously described importance of *g*_s_ for positively defining photosynthesis in wild barley (Fig. 3), it suggests that Steppe Jerusalem accessions may have evolved mechanisms that allow conserved water use by reducing stomatal opening with limited negative effects on photosynthesis. This means they do not have to elicit a significant iWUE response, in contrast to the Steppe Jerusalem accessions (Fig. 7e). In general, this highlights a degree of uncoupling between *g*_s_ and photosynthesis in the Desert Jordan accessions, which is unusual for a C_3_ species but highly attractive from the standpoint of improving the productivity of crops in water‐limited environments (Condon *et al*., 2004; Blum, 2005; Leakey *et al*., 2019).

## Competing interests

None declared.

## Author contributions

MB‐A and KS carried out data analyses. KS performed the targeted experiment. JNF led the field phenotyping with assistance from JR, CRGS, JW, GT, RLV and JM. EB, LDT and EF provided the germplasm and assisted with data analyses. TL, EF, JK and JNF designed the experiments. TL, EF and JK obtained funding. JNF wrote the paper with input from all authors. MB‐A and KS contributed equally to this work.

## Disclaimer

The New Phytologist Foundation remains neutral with regard to jurisdictional claims in maps and in any institutional affiliations.

## Supporting information


**Fig. S1** Location of common garden sites in 2021 and 2022.
**Fig. S2** Flow chart describing phenotyping activities.
**Fig. S3** Biplot highlight the positions of all accessions with respect to PC1 and PC2 from a PCA of all trait data. Accessions included in the targeted experiment are labelled.
**Fig. S4** Temperature in the glasshouse during the targetted experiment.
**Fig. S5** Photosynthetically active radiation (PAR) intensity in the glasshouse during the targeted experiment.
**Fig. S6** Boxplots showing mean monthly temperature for site of origin of all subpopulations.
**Fig. S7** (A) Daily maximum and minimum temperature during 2021 common garden experiment. (B) Daily maximum and minimum temperature during 2022 common garden experiment. (C) Differences in maximum and minimum temperatures between 2021 and 2022.
**Fig. S8** Daily water input (precipitation and irrigation) during (A) 2021 and (B) 2022 common garden experiment. (C) Average daily water input in 2021 and 2022.
**Fig. S9** Density plots showing trait variation for all traits not shown in Fig. 2.
**Fig. S10** Scatter plots showing correlations between years for all traits not shown in Fig. 2.
**Fig. S11** Pairwise trait correlations for all traits measured in 2021.
**Fig. S12** Pairwise trait correlations for all traits measured in 2022.
**Fig. S13** Boxplots showing trait variation across each subpopulation for all traits not shown in Fig. 5.
**Fig. S14** Boxplots showing variation in plasticity across each subpopulation for all traits not shown in Fig. 5.

**Table S1** List of accessions comprising both common garden experiments.
**Table S2** List of agronomic inputs for both common garden experiments.
**Table S3** Variance associated with each term for all models.
**Table S4** Heritabilities derived from year‐specific models.
**Table S5** Results from two‐way ANOVAs performed using glasshouse experiment data.
Please note: Wiley is not responsible for the content or functionality of any Supporting Information supplied by the authors. Any queries (other than missing material) should be directed to the *New Phytologist* Central Office.

## Data Availability

All data and R scripts used in this study are available on Zenodo (doi: 10.5281/zenodo.17121714).
