# Supplementary material for: Extensive photophysiological variation in wild barley is linked to environmental origin
Source: New Phytol. 2025 Nov 11;249(1):569–87. doi: 10.1111/nph.70659 (PMC12676107; doi:10.1111/nph.70659)
Supplement: Supplementary file 1 — Fig. S1 Location of common garden sites in 2021 and 2022. Fig. S2 Flow chart describing phenotyping activities. Fig. S3 Biplot highlight the positions of all accessions with respect to PC1 and PC2 from a PCA of all trait data. Accessions included in the targeted experiment are labelled. Fig. S4 Temperature in the glasshouse during the targetted experiment. Fig. S5 Photosynthetically active radiation (PAR) intensity in the glasshouse during the targeted experiment. Fig. S6 Boxplots showing mean monthly temperature for site of origin of all subpopulations. Fig. S7 (A) Daily maximum and minimum temperature during 2021 common garden experiment. (B) Daily maximum and minimum temperature during 2022 common garden experiment. (C) Differences in maximum and minimum temperatures between 2021 and 2022. Fig. S8 Daily water input (precipitation and irrigation) during (A) 2021 and (B) 2022 common garden experiment. (C) Average daily water input in 2021 and 2022. Fig. S9 Density plots showing trait variation for all traits not shown in Fig. 2. Fig. S10 Scatter plots showing correlations between years for all traits not shown in Fig. 2. Fig. S11 Pairwise trait correlations for all traits measured in 2021. Fig. S12 Pairwise trait correlations for all traits measured in 2022. Fig. S13 Boxplots showing trait variation across each subpopulation for all traits not shown in Fig. 5. Fig. S14 Boxplots showing variation in plasticity across each subpopulation for all traits not shown in Fig. 5. [file NPH-249-569-s002.pdf]

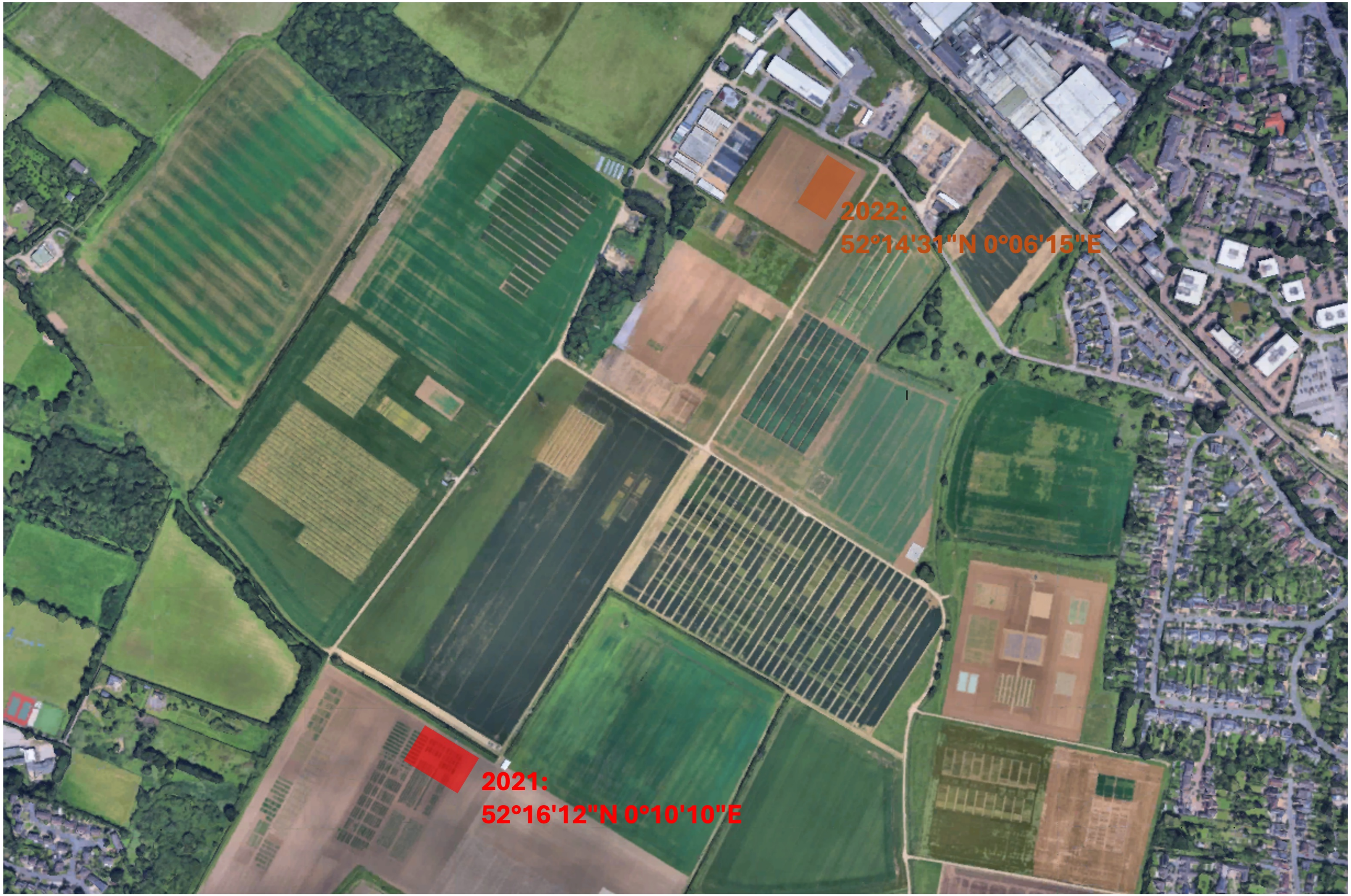

Supporting Figure S1. Common garden sites

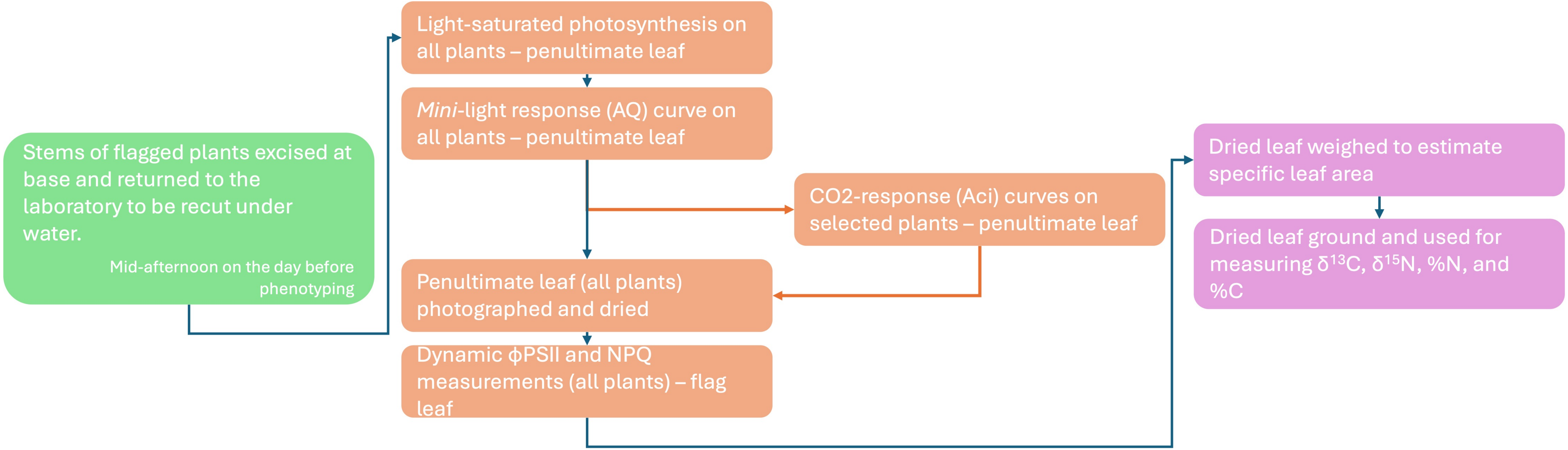

Supporting Figure S2. Phenotyping flow chart

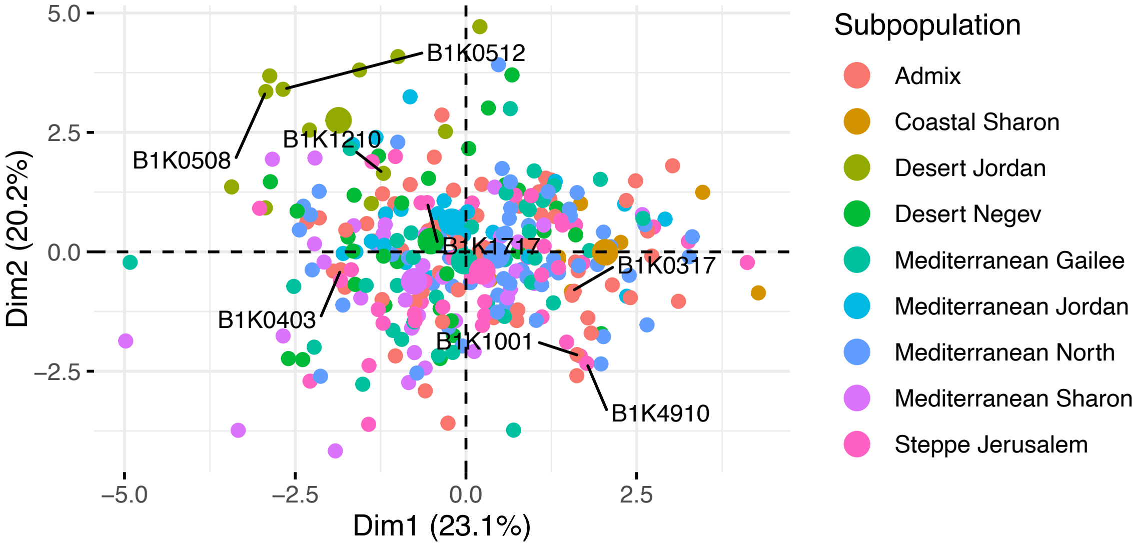

Supporting Figure S3. PCA biplot

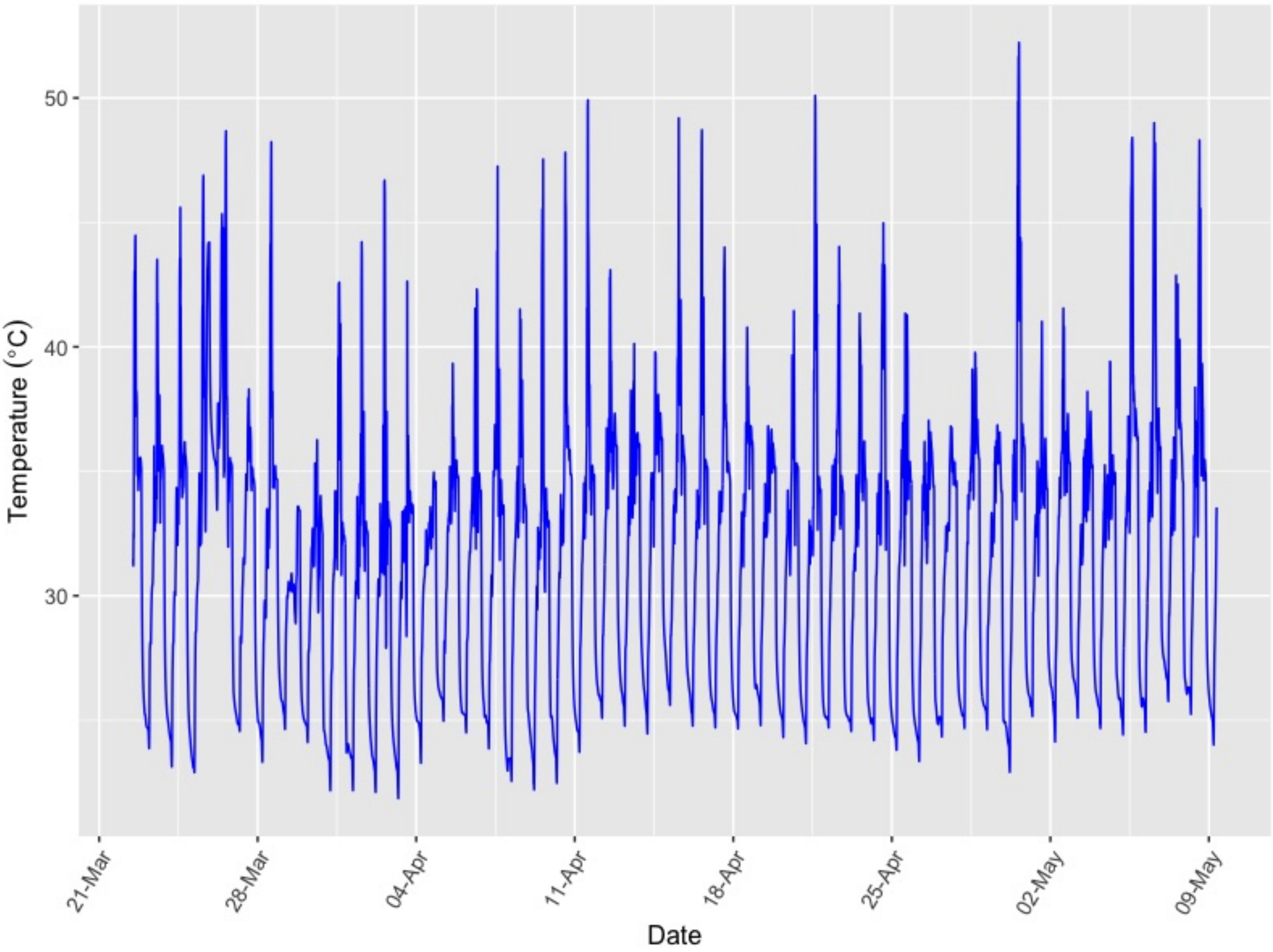

Supporting Figure S4. Temperature in the glasshouse

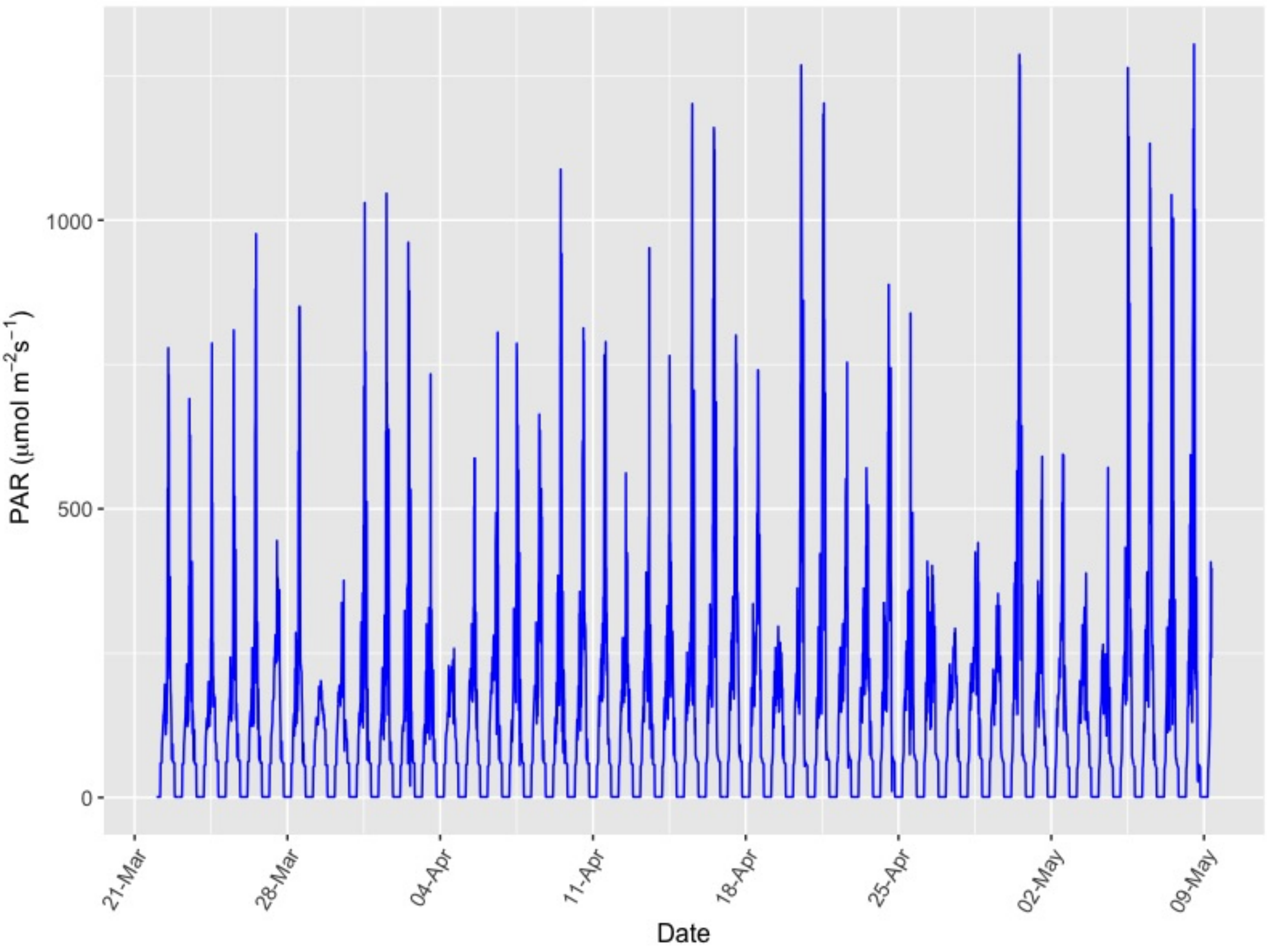

Supporting Figure S5. PAR in the glasshouse

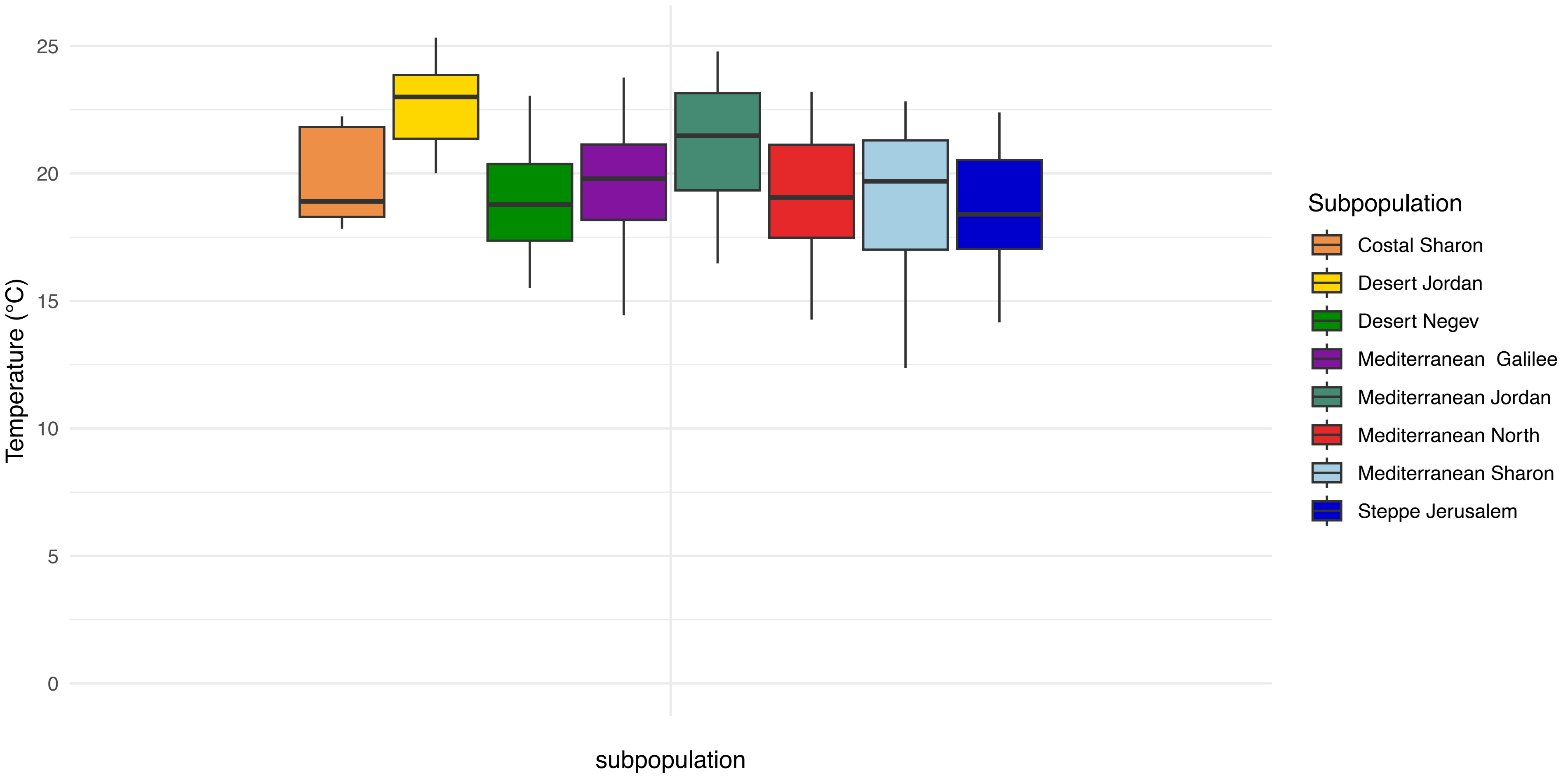

Supporting Figure S6. Boxplots showing mean monthly temperature for site of origin of all subpopulations

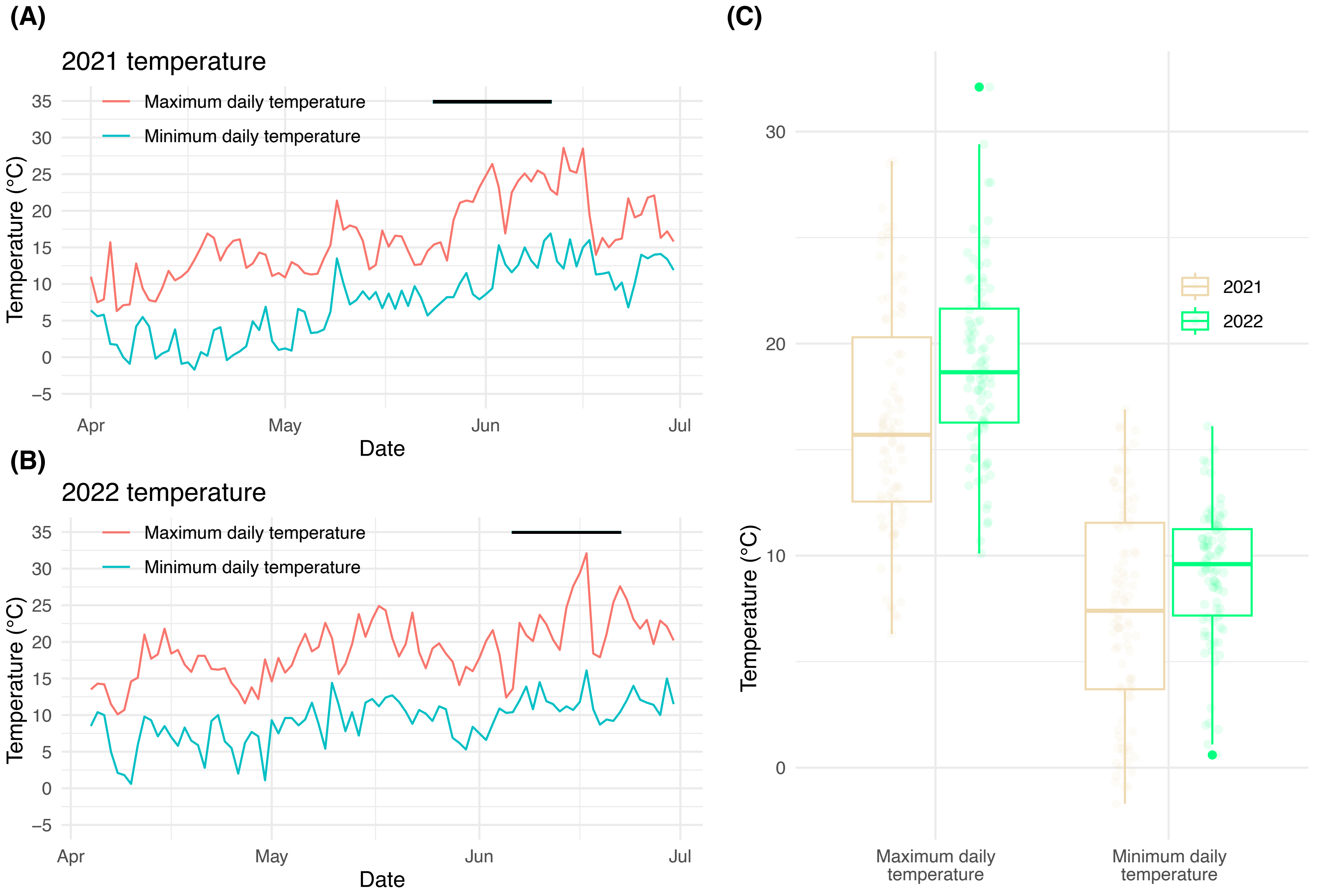

Supporting Figure S7. (A) Maximum and minimum temperature during 2021 common garden experiment. (B) Maximum and minimum temperature during 2022 common garden experiment. (C-D) Differences in temperature between 2021 and 2022.

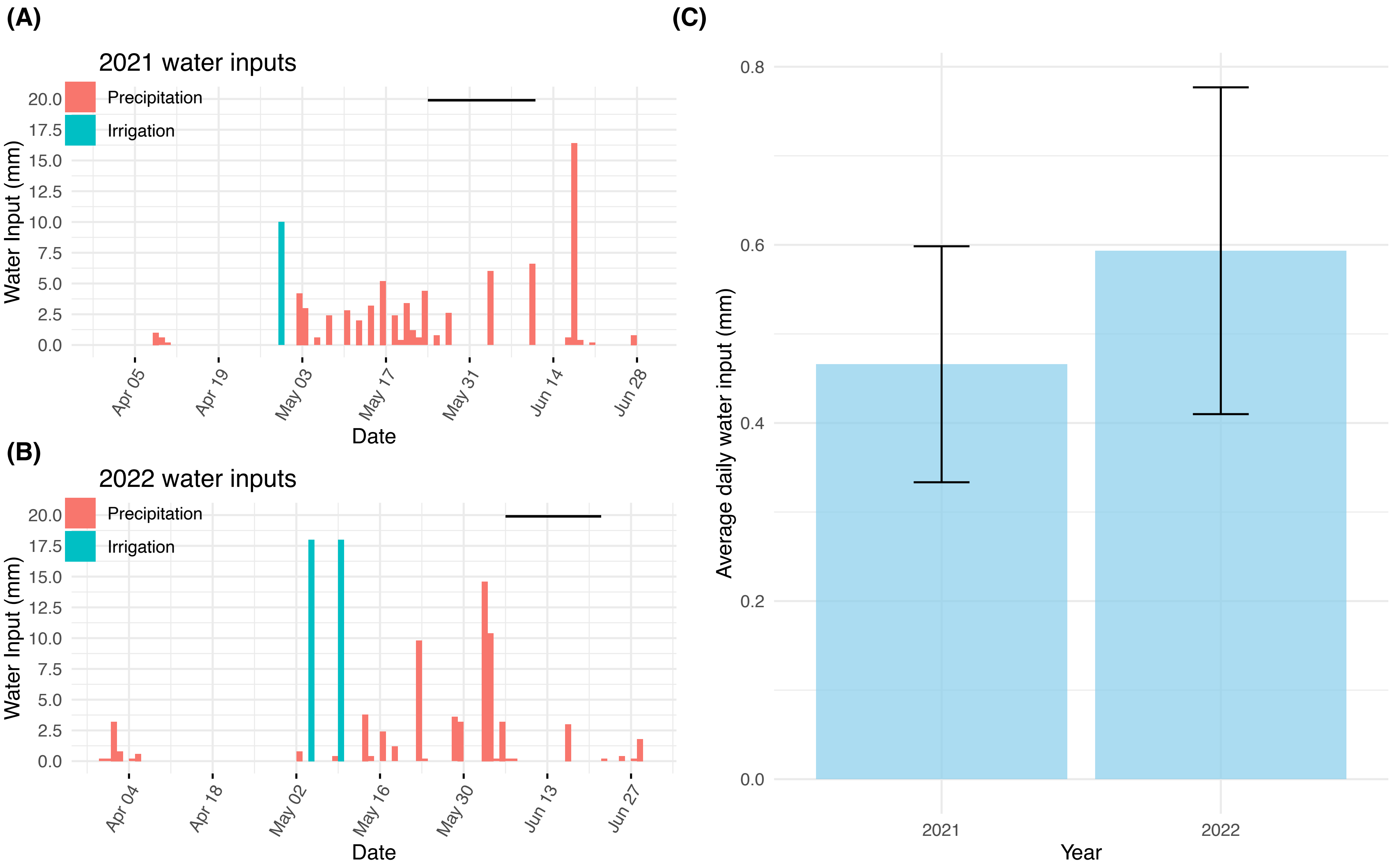

Supporting Figure S8. (A-B) Daily water input (precipitation and irrigation) during 2021 and 2022 common garden experiment. (C) Averagedaily water input in 2021 and 2022.

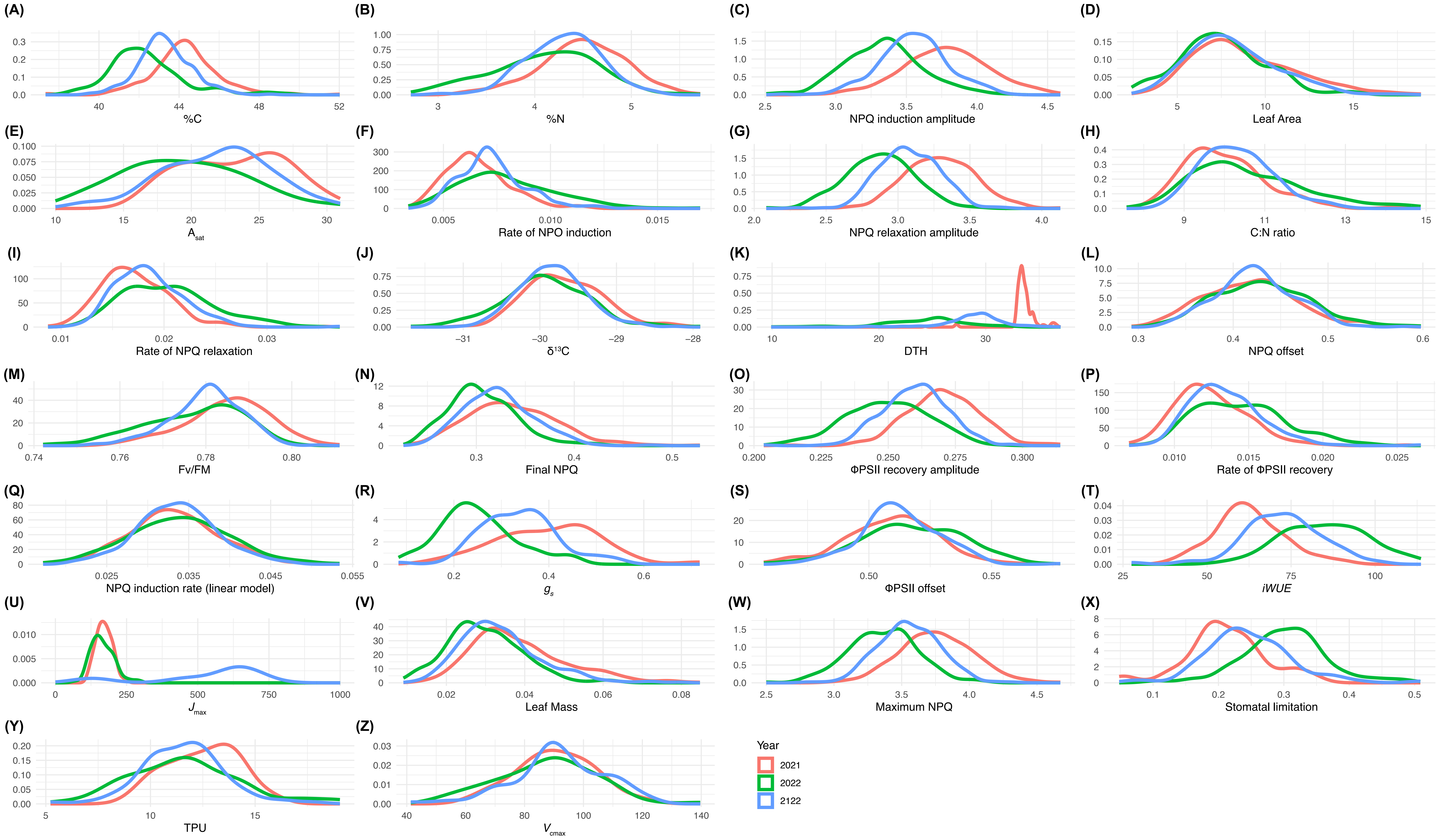

Supporting Figure S9. Density plots showing trait variation for traits not shown in Figure 2

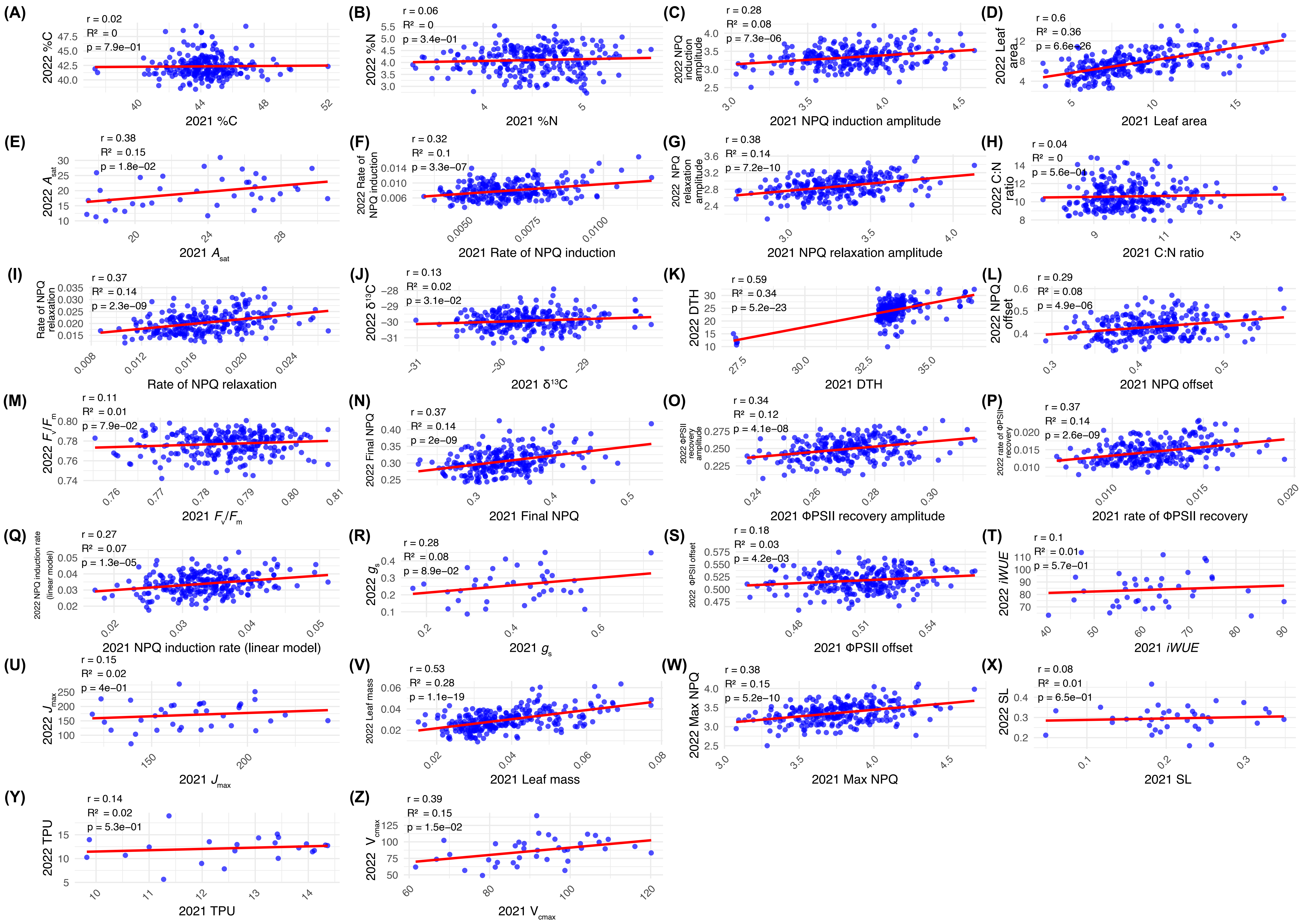

Supporting Figure S10. Scatter plots showing correlations between years for traits not shown in Figure 2.

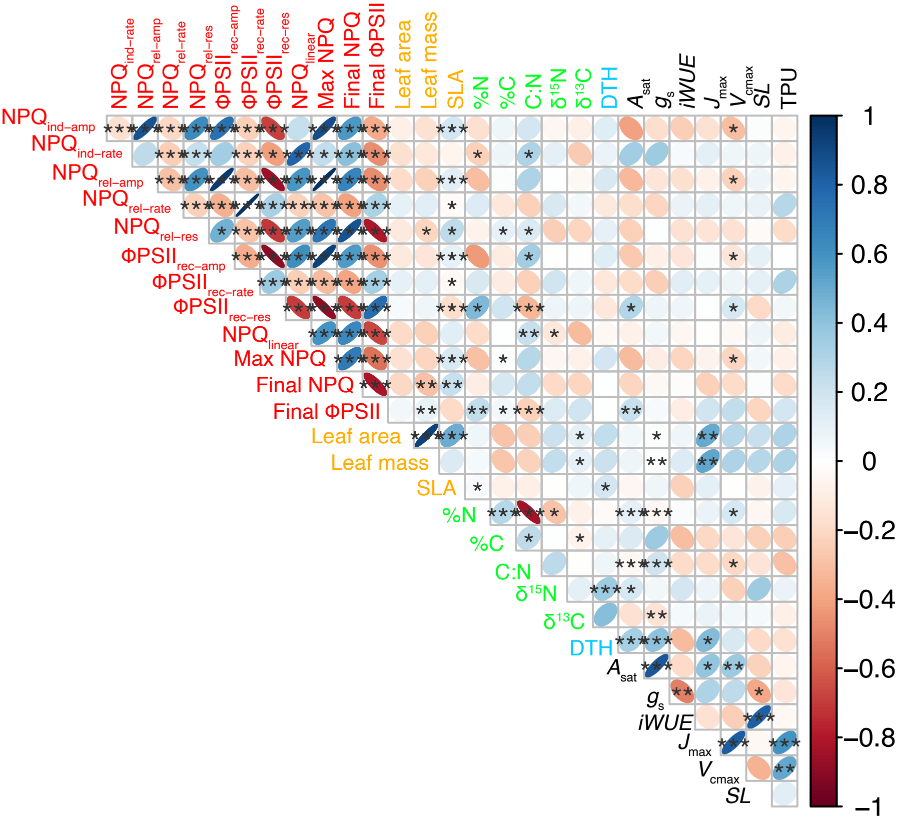

Supporting Figure S11. Pairwise trait correlations for 2021

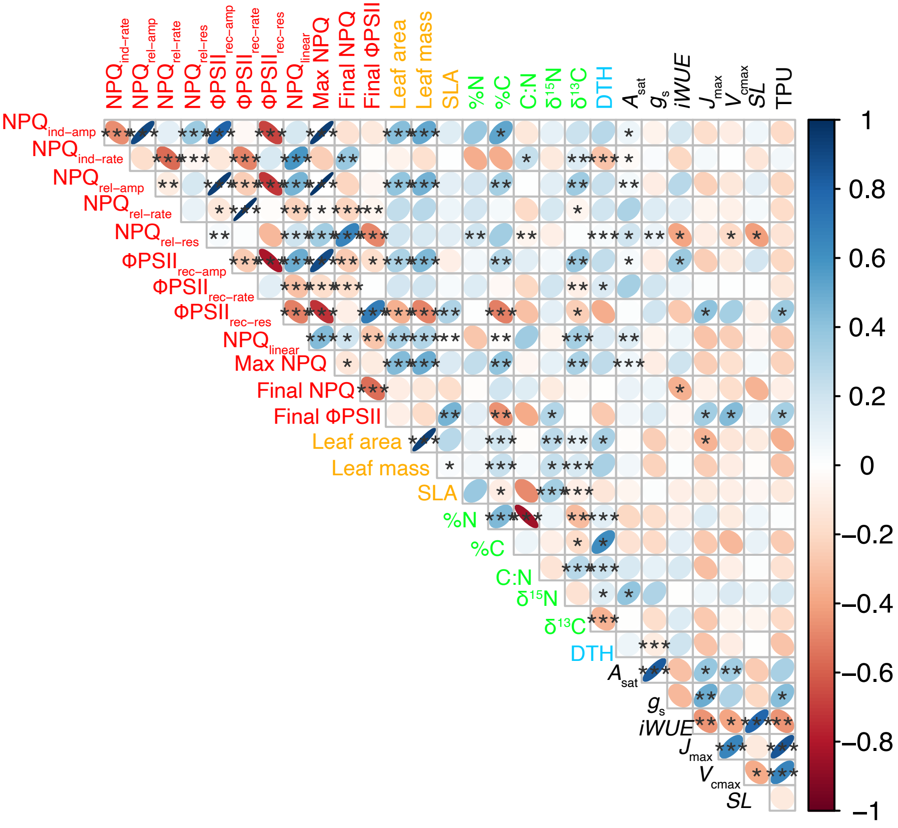

Supporting Figure S12. Pairwise trait correlations for 2022

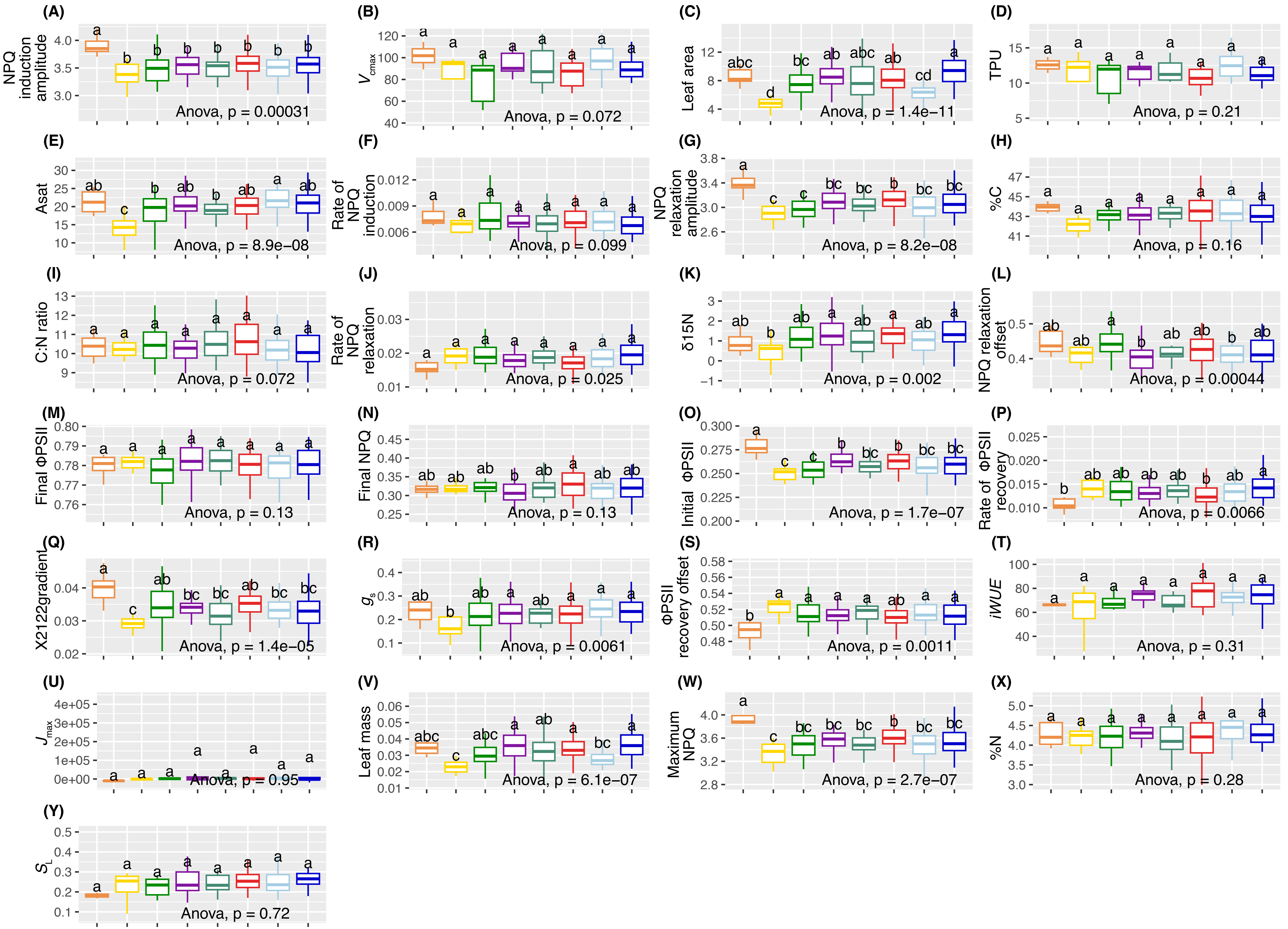

Supporting Figure S13. Boxplots for each subpopulation for traits not shown in Figure 5

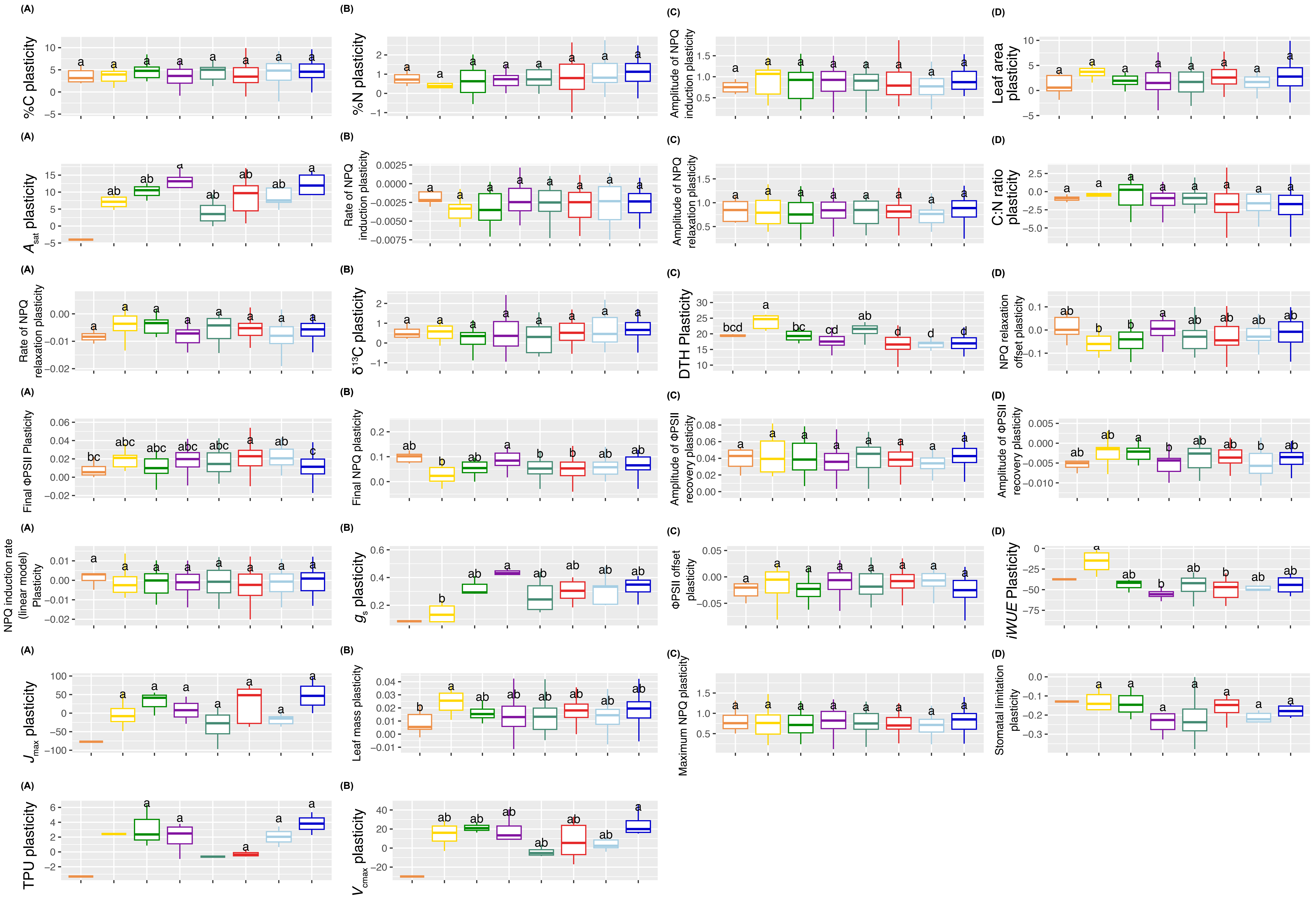

Supporting Figure S14. GxE plots for traits not shown in Figure 5
